# QSPR and QSTR analysis to explore pharmacokinetic and toxicity properties of antifungal drugs through topological descriptors

**DOI:** 10.1038/s41598-025-01522-0

**Published:** 2025-05-23

**Authors:** W. Tamilarasi, B. J. Balamurugan

**Affiliations:** Department of Mathematics, School of Advanced Sciences, Vellore Institute of Technology, Chennai Campus, Vandalur-Kelambakkam Road, Chennai, Tamil Nadu 600127 India

**Keywords:** Antifungal drugs, Topological indices, ADMET, LD_50_, Linear regression, QSPR analysis, Chemical biology, Computational biology and bioinformatics, Mathematics and computing

## Abstract

COVID-19 patients often develop serious fungal infections like Aspergillosis, Candidiasis, and Mucormycosis, which are treated with antifungal drugs like Amphotericin B, Posaconazole, and Isavuconazole. However, these treatments are often insufficient, leading researchers to explore drug combinations and analogs. In theoretical chemistry, a chemical molecule is converted into an isomorphic molecular graph, represented as G (V, E) by considering atom set V as vertices and bond set E as edges. Quantitative structure–activity/property/toxicity relationships (QSAR, QSPR, QSTR) modelling is a widely recognized discipline that correlates physicochemical and molecular descriptors with a drug’s bioactivity to predict its standard pharmacological properties. In this article, the aforementioned drugs, as well as some Amphotericin B analogs, with their properties, are considered for QSPR/QSTR analysis. The QSPR/QSTR analysis is carried out using linear regression between the computed topological indices (based on degree and neighbourhood degree sum) and pharmacokinetic (ADMET) and toxicity properties (LD_50_) of these drugs. The analysis reveals a strong correlation between the topological indices and the pharmacokinetic and toxicity properties of the drugs and their analogs. These insights are crucial for advancing more effective antifungal treatments, especially for COVID-19-related infections.

## Introduction

Since COVID-19 affects the immune system, it raises the risk of developing a fungal infection, and several antiviral medications and steroids used to treat COVID-19 can make the body less resistant to fungus. Aspergillosis, an infection caused by the fungus Aspergillus, is increasingly being identified in immunocompromised patients and those suffering with COVID-19 with severe respiratory infections. Pulmonary aspergillosis associated to COVID-19 been described in several recent papers^1,2^. Mucormycosis commonly known as Black fungus disease is a rare fungal infection caused by mucoromycetes^3^. It has been speculated that this disease is in close association with the recently emerged COVID-19. COVID-19 pandemic has paved way for a myriad of manifestations and complications including several life threatening secondary complications caused by fungal infections like Mucormycosis^4^. Therapeutic management of this disease displayed good result with amphotericin B^5^. The initial stage of this pandemic have opened up avenues for many repurposed medicines and natural products for prophylaxis^6^. The ability of many drug candidates to efficiently interact with the key enzymes of the fungus is being analyzed computationally with molecular docking, MM-GBSA and Molecular Dynamics (MD) simulation assessment^7^. High risk of infection was observed in patients under steroid medication and diabetic^8,9^. Patients with COVID-19 who developed candidemia were more likely to have immediate risk factors connected to COVID-19 treatment, like immune system suppressing drugs, and less likely to have certain underlying illnesses and procedures frequently associated with candidemia^10^.

Antifungal medications are essential for the treatment of Aspergillosis, Candidiasis, and Mucormycosis. Among these, Posaconazole, Amphotericin B and Isavuconazole are frequently employed to manage Mucormycosis. A recent review^11^ has revealed the pharmacological and pharmacokinetic properties, structures, drug-target interactions, stability etc., for Amphotericin B, a vital medication used to treat a number of invasive fungal diseases. The pharmacokinetics, pharmacodynamics, toxicity and clinical studies on another drug Posaconazole was also reported with low hepatotoxicity and cardiotoxicity^12^. Recently, a novel antifungal medication, Isavuconazole, received approval for treating aspergillosis and Mucormycosis, in addition to its established application for CNS and fungal infections that invade the body. The latter is accessible in a water-soluble intravenous formulation, characterized by outstanding bioavailability and consistent pharmacokinetics^13^. The growing prevalence of antifungal diseases represents a substantial challenge for the pharmaceutical sector in its endeavour to create new drugs featuring superior pharmacological characteristics when compared to those presently available. Moreover, currently there is no medication to halt or prevent such fungal diseases. Hence, researchers are exploring combinations of existing medications and their derivatives. In this paper Posaconazole, Isavuconazole and some analogs of Amphotericin B, which are potentially active towards the treatment of fungal diseases are investigated via chemical graph theory to correlate the pharmacokinetic and pharmacodynamics properties with standard drugs.

In chemical graph theory, topological index is considered as a powerful and useful descriptor for molecular structures of chemical compounds. In the development of QSPR/QSAR, topological indices are utilized to correlate chemical structures with physical, chemical, and biological activities, as well as additional features like ADMET (absorption, distribution, metabolism, excretion, and toxicity). Many studies have highlighted a robust inherent connection between the chemical attributes of drugs, such as boiling point, flash point, and enthalpy, and their molecular structures. Topological indices calculated from these chemical structures can assist researchers in gaining a deeper comprehension of the interconnectedness between physical characteristics, chemical reactivity, and biological activity. The examination of topological indices within the chemical structure of drugs can serve as a valuable substitute for chemical experiments, offering a theoretical foundation for drug manufacturing processes. There are many topological indices for graphs based on degree, distance, eccentricity, etc. Herein, topological indices related to degree and neighbourhood degree sum^14^ are generated for few antifungal drugs. Computation of topological indices from their molecular formula will be a tedious process for large graphs. To make the calculations easier there exist many algebraic polynomials from which some indices are deduced through differentiation and integration. The most frequently used polynomials for deriving certain degree-based and neighbourhood degree sum-based topological indices are $M$-polynomial and $NM$-polynomial^14^.

The extensive computational evaluation of topological indices has led to the development of various distance-based and degree-based algebraic polynomials, including the Zagreb polynomial^15^, Hosoya polynomial^16^, Forgotten polynomial^17^, and Schultz polynomial^18^, among others. The recently evolved $M$-polynomial^19^ and $NM$-polynomial^14^ can efficiently generate various indices based on degree and neighbourhood degree sum^20^ respectively. The $M$-polynomial^19^ of a graph G is defined as follows:

$$M\left( {G;x,y} \right) = \mathop \sum \limits_{\delta \le i \le j \le \Delta } e_{i,j} x^{i} y^{j}$$

where $e_{i,j} , i,j \ge 1,$ is a number of edges $uv \in E$(G) and $\left( {d_{u} ,d_{v} } \right) = \left( {i,j} \right)$, $d_{u}$ and $d_{v}$ represent the degree of the vertices $u$ and $v$ respectively and $\left( {\delta ,\Delta } \right) = \left( {\min d_{v} ,\max d_{v} } \right)$ where $u,v \in V$(G).

Similarly, $NM$-polynomial is used to generate indices based on neighbourhood degree sum and is defined as follows.

$$NM\left( {G;x,y} \right) = \mathop \sum \limits_{\delta \le i \le j \le \Delta } e_{i,j}^{*} x^{i} y^{j}$$

where $e_{i,j}^{*} , i,j \ge 1,$ is a number of edges $uv \in E$(G) and $\left( {nd_{u} ,nd_{v} } \right) = \left( {i,j} \right)$, $nd_{u}$ and $nd_{v}$ represent the neighbourhood degree sum of the vertices $u$ and $v$ respectively and $\left( {\delta ,\Delta } \right) = \left( {\min d_{v} ,\max d_{v} } \right)$ where $u,v \in V$(G).

The degree based and neighbourhood degree sum based topological indices with their M & NM—polynomials are presented in Table 1.

**Table 1:** Indices formulae and their mathematical derivation from M & NM—polynomial.

Topological index	*f (d*_*u*_*, d*_*v*_*)*	*f (x, y)* = *M (G; x, y)* or* NM (G; x, y)*
First Zagreb index $M_{1} \left( G \right)$ Third version Zagreb index $NM_{1} \left( G \right)$	$\mathop \sum \limits_{uv \in E\left( G \right)} \left( {d_{u} + d_{v} } \right)$	$\left( {D_{x} + D_{y} } \right)\left( {f\left( {x,y} \right)} \right)|_{x = y = 1}$
Second Zagreb index $M_{2} \left( G \right)$ Neighbourhood second Zagreb index $NM_{2} \left( G \right)$	$\mathop \sum \limits_{uv \in E\left( G \right)} \left( {d_{u} d_{v} } \right)$	$\left( {D_{x} D_{y} } \right)\left( {f\left( {x,y} \right)} \right)|_{x = y = 1}$
Second modified Zagreb index $m{{M_{2} }} \left( G \right)$ Neighbourhood second modified Zagreb index $Nm{{M_{2} }} \left( G \right)$	$\mathop \sum \limits_{uv \in E\left( G \right)} \frac{1}{{d_{u} d_{v} }}$	$\left( {I_{x} I_{y} } \right)\left( {f\left( {x,y} \right)} \right)|_{x = y = 1}$
Redefined third Zagreb index ReZ $G_{3} \left( G \right)$ Third NDe index $ND_{3} \left( G \right)$	$\mathop \sum \limits_{uv \in E\left( G \right)} d_{u} d_{v} \left( {d_{u} + d_{v} } \right)$	$\left( {D_{x} D_{y} } \right){ }\left( {D_{x} + D_{y} } \right)\left( {f\left( {x,y} \right)} \right)|_{x = y = 1}$
Forgotten topological index $F\left( G \right)$ Neighbourhood Forgotten topological index $NF\left( G \right)$	$\mathop \sum \limits_{uv \in E\left( G \right)} \left( {d_{u}^{2} + d_{v}^{2} } \right)$	$\left( {D_{x}^{2} + D_{y}^{2} } \right)\left( {f\left( {x,y} \right)} \right)|_{x = y = 1}$
Symmetric Division degree index $SDD\left( G \right)$ Fifth NDe index $ND_{5} \left( G \right)$	$\mathop \sum \limits_{uv \in E\left( G \right)} \frac{{d_{u}^{2} + d_{v}^{2} }}{{d_{u} d_{v} }}$	$\left( {I_{y} D_{x} + I_{x} D_{y} } \right)\left( {f\left( {x,y} \right)} \right)|_{x = y = 1}$
Harmonic index $H\left( G \right)$ Neighbourhood Harmonic index $NH\left( G \right)$	$\mathop \sum \limits_{uv \in E\left( G \right)} \frac{2}{{d_{u} + d_{v} }}$	$\left( {2I_{x} J} \right)\left( {f\left( {x,y} \right)} \right)|_{x = 1}$
Inverse sum indeg index $I\left( G \right)$ Neighbourhood inverse sum index $NI\left( G \right)$	$\mathop \sum \limits_{uv \in E\left( G \right)} \frac{{d_{u} d_{v} }}{{d_{u} + d_{v} }}$	$\left( {I_{x} JD_{x} D_{y} } \right)\left( {f\left( {x,y} \right)} \right)|_{x = 1}$

where, $$\begin{gathered} D_{x} = x\left( {\frac{{\partial \left( {f\left( {x,y} \right)} \right)}}{\partial x}} \right), D_{y} = y\left( {\frac{{\partial \left( {f\left( {x,y} \right)} \right)}}{\partial y}} \right), I_{x} = \mathop \smallint \limits_{0}^{x} \frac{{f\left( {t,y} \right)}}{t}dt, I_{y} = \mathop \smallint \limits_{0}^{y} \frac{{f\left( {x,t} \right)}}{t}dt, \hfill \\ J\left( {f\left( {x,y} \right)} \right) = f\left( {x,x} \right) , Q_{k} \left( {f\left( {x,y} \right)} \right) = x^{k} f\left( {x,y} \right) \hfill \\ \end{gathered}$$

The QSTR model have found extensive application in evaluating the safety of chemicals and pharmaceuticals. They can be employed to forecast the toxicity or activity of a substantial quantity of untested chemical compounds. Toxicity is assessed by means of LD_50_ (Lethal Dose), which serves as a standardized measure for quantifying and comparing the toxicity levels of various chemicals. Though the existing molecular docking simulations are being adopted to investigate the drug-biomolecular interactions, these correlation studies will be of great help to investigate and compare the drug-ability based on certain parameters. Herein the QSPR and QSTR analysis of some indices based on degree and neighbourhood degree sum were investigated for some antifungal drugs used for the treatment of fungal disease following $M$-polynomial and $NM$-polynomial^20^ approaches.

Our research on topological descriptors of isomorphic molecular structures is based on mathematical and graph-theoretical principles, along with statistical analysis, without involving any laboratory experiments. The topological descriptors, derived from molecular structures, are used to analyse the ADMET properties of molecules. There are other descriptors available in literature such as physico-chemical, quantum mechanical, geometrical descriptors and Morgan fingerprints. The physico-chemical descriptors are numerical values that represent the physical and chemical properties of molecules obtained through laboratory experiments^21^. Quantum mechanical descriptors use quantum chemistry methods such as Density Functional Theory (DFT) and ab initio calculations (first-principles calculations) to predict the electronic properties of molecules^22^. Geometrical descriptors are molecular descriptors that quantify the three-dimensional (3D) shape, size, and spatial arrangement of atoms in a molecule^23^. Morgan fingerprint descriptors are a type of molecular descriptor that encode the structural and topological features of a molecule using a circular hashing algorithm. They are widely used in cheminformatics for molecular similarity analysis, virtual screening, and drug discovery, as they efficiently capture atom connectivity and substructural patterns^24^. Physico-chemical, quantum mechanical, geometrical descriptors, and Morgan fingerprints all fall outside our methodological framework. Thus, topological descriptors are the best suitable choice for QSPR analysis to predict the ADMET properties of antifungal drugs.

## Literature review

The topological indices are mostly characterized by distance-based, degree-based and spectral-based concept. Harold Wiener in 1947, introduced the first topological index called Wiener index and is used to predict physical properties of alkanes^25^. Gutman and Trinajstic^26^ in 1972 introduced the first and second Zagreb indices and they stated that these indices are useful in the study of anti-inflammatory activities of certain chemical instances. The Zagreb index is used in the study of molecular complexity, heterosystems, ZE-isomerism and many researches use this index for QSPR and QSAR studies. Favaron et al. in^27^, established the relation between harmonic index and eigen values of molecular graphs. The Symmetric Division degree index (SDD)^28^ is one of the 148 discrete Adriatic indices that proved to be a valuable index in the QSPR / QSAR studies. SDD index has strong correlation ability in the prediction of total surface area of polychlorobiphenyls^29^. Ghorbani et al. in^30^ defined third version of Zagreb index and found that this index and the acentric factor and entropy for octane isomers have a strong correlation.

Using degree based topological indices, some anticancer and antiviral drugs properties were investigated with QSPR analysis for some diseases like cancer, COVID-19, dengue etc.^31–46^. Recently in^47,48^ the QSPR model for ADMET properties of drugs against Omicron variant with some degree based topological indices via $M$-polynomial and Zika virus drugs using Revan indices were investigated. Sourav Mondal et al.^20^ utilized degree-based and neighborhood degree sum-based topological indices to investigate antiviral drugs used for the treatment of COVID-19 patients. Similarly, M.C. Shanmukha et al.^31^ focused on degree-based topological indices applied to anticancer drugs through QSPR analysis. Syed Ajaz K. Kirmani et al.^33^ explored several degree and neighborhood degree sum-based indices for antiviral drugs treating COVID-19 using M-polynomial and NM-polynomial methods. Additionally, Syed Ahtsham Ul Haq Bokhary et al.^36^ analyzed well-known degree-based indices on chemical structures of medicines for breast cancer treatment. Havare^37^ applied M-polynomial and NM-polynomial approaches to compute degree and neighborhood degree sum-based indices for novel drugs used in cancer treatments.

While these studies demonstrate the utility of degree-based and neighborhood degree sum-based indices in drug analysis, they are limited in scope, focusing predominantly on antiviral or anticancer drugs. Furthermore, the QSPR analyses in existing studies primarily address the physicochemical properties of drugs without exploring ADMET (Absorption, Distribution, Metabolism, Excretion, and Toxicity) properties. In this article, we address these gaps by investigating both QSPR and QSTR analyses for antifungal drugs using degree and neighborhood degree sum-based indices. This approach has not been explored in the existing literature. Moreover, our research uniquely includes QSPR and QSTR analysis for ADMET properties, which are crucial in drug discovery and development, thereby providing a novel contribution to the field.

## Methodology and main results

**Fig. 1:**
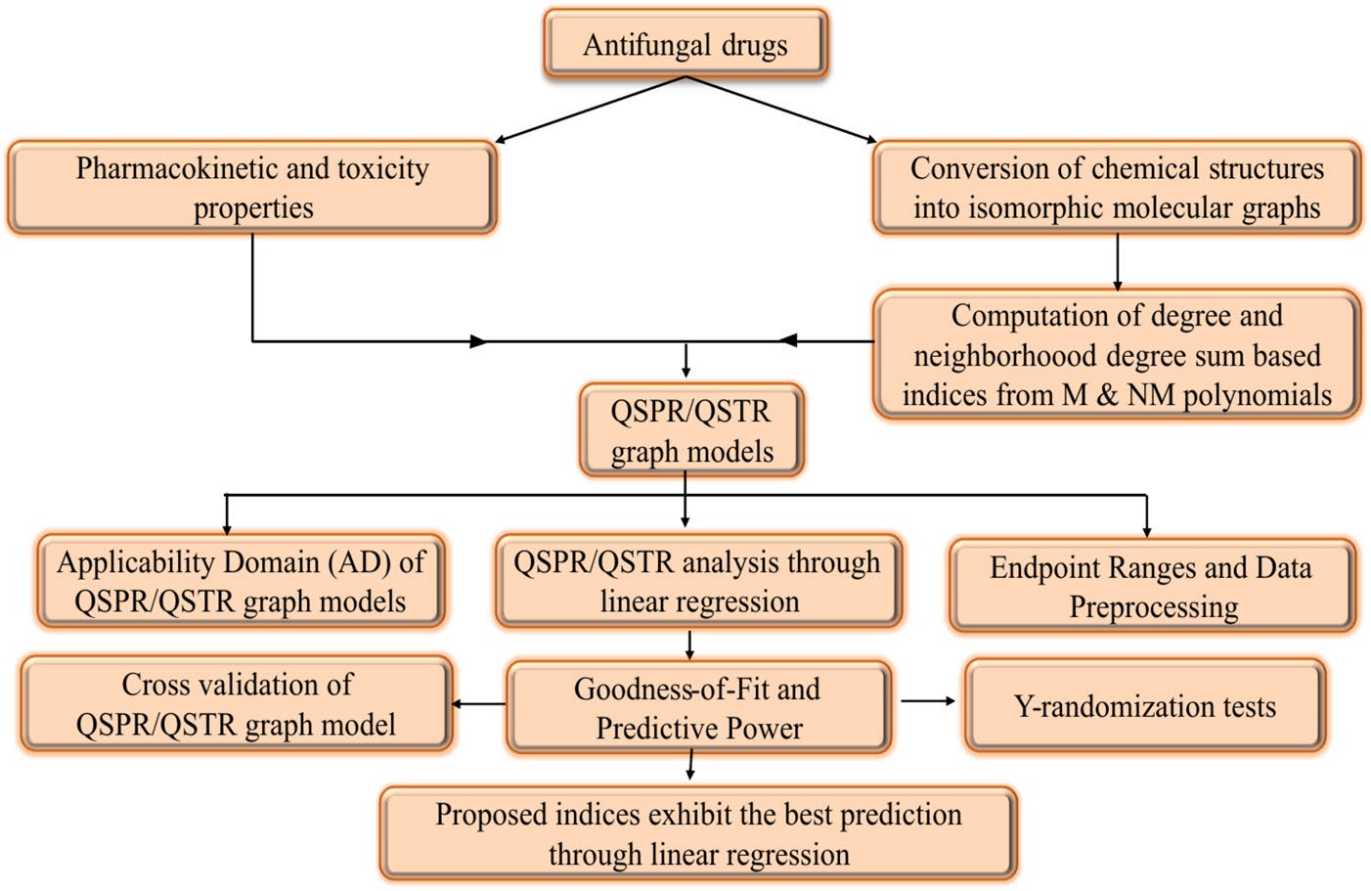
Workflow of the article.

This section introduces the concept of isomorphic molecular graphs, as first proposed by Tamilarasi et al. in^49^. The isomorphic molecular graphs are used to explore the ADMET and toxicity properties of antifungal drugs. Additionally, the degree and neighbourhood degree sum-based indices are computed for the isomorphic molecular graphs of Amphotericin B, Posaconazole, Isavuconazole, and analogs of Amphotericin B. The research work reported in this article for QSPR/QSTR regression analysis to predict the properties of the aforementioned antifungal drugs, is summarized in the flowchart shown in Fig. 1.

### Motivation

Wiener^25^ introduced two topological descriptors called Wiener index and Polarity index. Using these indices, Wiener predicted the boiling point of alkanes, where the chemical structure of alkanes contains only the single bond. Computing the Wiener index for the molecular graph of a chemical compound formed by considering double and triple bonds as a single edge does not affect the index value, as the Wiener index is a distance-based topological descriptor. Moreover, Wiener, excluded hydrogen atoms to simplify molecular graphs by focusing on atom connectivity. His index captures key structural features b y measuring distances between atom pairs, avoiding the added complexity of hydrogen atoms. Later, many studies have been carried out by researchers on various topological indices for the prediction of properties of the chemical compounds by considering hydrogen depleted molecular graph and the double bond and triple bond as a single edge. In^50^ Ivan Gutman and Oskar E. Polansky introduced a concept known as complete molecular graph, including all hydrogen atoms in the molecular framework but still considering double and triple bonds as a single edge.

Many researchers construct molecular graphs by converting double and triple bonds into a single edge instead of multiple edges. However, this approach is not acceptable from a chemist’s perspective and goes against graph theory principles. In our research, we compute the degree-based topological indices of molecular graphs and these indices are mainly computed by the degree of atoms. Hence, in this article, the chemical structures are converted into isomorphic molecular graphs by incorporating, double bonds as two parallel edges, triple bonds as three parallel edges and hydrogen and other atoms are preserved in their adjacency. These isomorphic molecular graphs will stimulate accurate representation of chemical sructure of a molecule.This approach preserves the unique structural characteristics of the molecule, allowing for accurate comparison and analysis.

#### Definition 1

Let $M$ be a molecular structure of a chemical molecule. The isomorphic molecular graph of $M$ is a graph $G\left( {V, E} \right)$ formed by considering atom set $V$ as vertices and bond set $E$ as edges. The double, triple bonds in $M$ are considered as two and three parallel edges respectively and vertex set $V$ ]includes hydrogen atoms in isomorphic molecular graph $G\left( {V, E} \right)$.

**Fig. 2:**
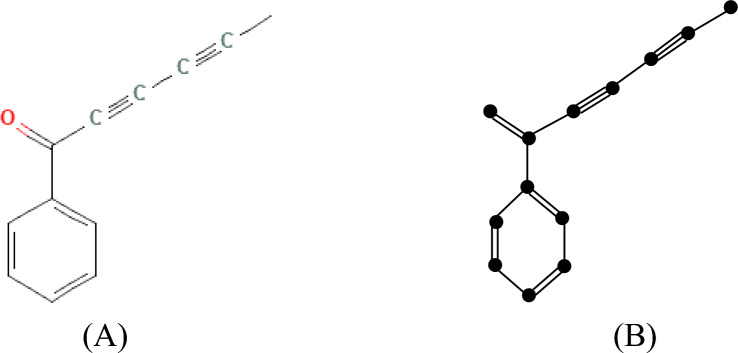
(**A**) Chemical structure and (**B**) Isomorphic chemical graph of Capillin.

The following Fig. 2A,B depicts the chemical structure and isomorphic molecular graph of Capillin respectively.

The expressions of $M$-Polynomial and $NM$-Polynomial for the isomorphic molecular graphs of Amphotericin B, Posaconazole, Isavuconazole, and analogs of Amphotericin B were computed using degree and neighbourhood degree sum-based edge partition method in the following theorems. The chemical structure of the aforementioned antifungal medications are sourced from “www.pubchem.ncbi.nlm.nih.gov”.

#### **Theorem 1**

*Let* $P\left( {V,E} \right)$ *be the isomorphic molecular graph of Posaconazole, then the* $M$ *and* $NM$ *polynomials of the molecular graph* $P$ *are*.

$$M\left( {P;x,y} \right) = 2xy^{2} + 2xy^{3} + xy^{4} + 4x^{2} y^{2} + 10x^{2} y^{3} + 6x^{2} y^{4} + 24x^{3} y^{3} + 19x^{3} y^{4} + 3x^{4} y^{4}$$

$$\begin{aligned} M\left( {P;x,y} \right) & = 2x^{2} y^{4} + 2x^{3} y^{6} + x^{4} y^{6} + 4x^{4} y^{8} + 2x^{5} y^{5} + 4x^{5} y^{6} \\ & \quad + 4x^{5} y^{8} + 8x^{6} y^{6} + 4x^{6} y^{7} + 6x^{6} y^{8} + 2x^{6} y^{9} + 2x^{6} y^{10} \\ & \quad + x^{6} y^{11} + 5x^{7} y^{7} + 4x^{7} y^{8} + 7x^{7} y^{9} + 2x^{7} y^{10} + x^{7} y^{11} \\ & \quad + x^{8} y^{8} + 2x^{8} y^{9} + 2x^{8} y^{10} + 3x^{8} y^{11} + x^{9} y^{11} + x^{10} y^{11} \\ \end{aligned}$$

#### Proof

**Fig. 3:**
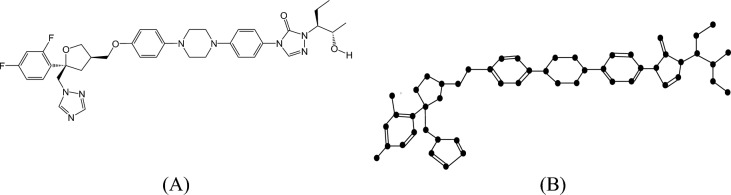
(**A**) Chemical structure and (**B**) Isomorphic molecular graph of Posaconazole.

The molecular structure and isomorphic graph representation of Posaconazole are depicted in Fig. 3A,B respectively. Let $P$ be the isomorphic chemical graph of Posaconazole with 52 vertices (atoms) and 71 edges (bonds). Let $E_{i,j} = \left\{ {uv \in E\left( G \right):d_{u} = i, d_{v} = j} \right\}$ and $\left| {E_{i,j} } \right| =$ $e_{i,j}$. From Fig. 3B, the degree-based edge partitions are $e_{1,2} = 2,e_{1,3} = 2, e_{1,4} = 1, e_{2,2} = 4, e_{2,3} = 10, e_{2,4} = 6, e_{3,3} = 24,e_{3,4} = 19,e_{4,4} = 3$. The $M$-Polynomial for the drug Posaconazole is obtained as follows:

$$\begin{aligned} M\left( {P;x,y} \right) & = \mathop \sum \limits_{\delta \le i \le j \le \Delta } e_{i,j} x^{i} y^{j} \\ & = e_{1,2} xy^{2} + e_{1,3} xy^{3} + e_{1,4} xy^{4} + e_{2,2} x^{2} y^{2} + e_{2,3} x^{2} y^{3} + e_{2,4} x^{2} y^{4} + e_{3,3} x^{3} y^{3} + e_{3,4} x^{3} y^{4} + e_{4,4} x^{4} y^{4} \\ & = 2xy^{2} + 2xy^{3} + xy^{4} + 4x^{2} y^{2} + 10x^{2} y^{3} + 6x^{2} y^{4} + 24x^{3} y^{3} + 19x^{3} y^{4} + 3x^{4} y^{4} \\ \end{aligned}$$

Similarly, let $E_{i,j}^{*} = \left\{ {uv \in E\left( G \right):nd_{u} = i, nd_{v} = j} \right\}$ and $\left| {E_{i,j} } \right| =$ $e_{i,j}^{*}$. Thus, from Fig. 3B, the neighbourhood degree sum-based edge partitions are

$$e_{2,4}^{*} = 2,e_{3,6}^{*} = 2,e_{4,6}^{*} = 1,e_{4,8}^{*} = 4,e_{5,5}^{*} = 2,e_{5,6}^{*} = 4,e_{5,8}^{*} = 4,e_{6,6}^{*} = 8,e_{6,7}^{*} = 4,$$

$$e_{6,8}^{*} = 6,e_{6,9}^{*} = 2,e_{6,10}^{*} = 2,e_{6,11}^{*} = 1,e_{7,7}^{*} = 5,e_{7,8}^{*} = 4,e_{7,9}^{*} = 7,e_{7,10}^{*} = 2,e_{7,11}^{*} = 1,$$

$$e_{8,8}^{*} = 1,e_{8,9}^{*} = 2,\;e_{8,10}^{*} = 2,e_{8,11}^{*} = 3,e_{9,11}^{*} = 1,e_{10,11}^{*} = 1.$$

Similarly, the $NM$-Polynomial for the drug Posaconazole is obtained as follows:

$$\begin{aligned} NM\left( {P;x,y} \right) & = \mathop \sum \limits_{\delta \le i \le j \le \Delta } e_{i,j}^{*} x^{i} y^{j} \\ & = e_{2,4}^{*} x^{2} y^{4} + e_{3,6}^{*} x^{3} y^{6} + e_{4,6}^{*} x^{4} y^{6} + e_{4,8}^{*} x^{4} y^{8} + e_{5,5}^{*} x^{5} y^{5} \\ & \quad + e_{5,6}^{*} x^{5} y^{6} + e_{5,8}^{*} x^{5} y^{8} + e_{6,6}^{*} x^{6} y^{6} + e_{6,7}^{*} x^{6} y^{7} + e_{6,8}^{*} x^{6} y^{8} + e_{6,9}^{*} x^{6} y^{9} \\ & \quad + e_{6,10}^{*} x^{6} y^{10} + e_{6,11}^{*} x^{6} y^{11} + e_{7,7}^{*} x^{7} y^{7} + e_{7,8}^{*} x^{7} y^{8} + e_{7,9}^{*} x^{7} y^{9} + e_{7,10}^{*} x^{7} y^{10} \\ & \quad + e_{7,11}^{*} x^{7} y^{11} + { }e_{8,8}^{*} x^{8} y^{8} + e_{8,9}^{*} x^{8} y^{9} + e_{8,10}^{*} x^{8} y^{10} + e_{8,11}^{*} x^{8} y^{11} \\ & \quad + e_{9,11}^{*} x^{9} y^{11} + e_{10,11}^{*} x^{10} y^{11} \\ & = 2x^{2} y^{4} + 2x^{3} y^{6} + x^{4} y^{6} + 4x^{4} y^{8} + 2x^{5} y^{5} + 4x^{5} y^{6} + 4x^{5} y^{8} \\ & \quad + 8x^{6} y^{6} + 4x^{6} y^{7} + 6x^{6} y^{8} + 2x^{6} y^{9} + 2x^{6} y^{10} + x^{6} y^{11} + 5x^{7} y^{7} + 4x^{7} y^{8} + 7x^{7} y^{9} + 2x^{7} y^{10} \\ & \quad + x^{7} y^{11} + x^{8} y^{8} + 2x^{8} y^{9} + 2x^{8} y^{10} + 3x^{8} y^{11} + x^{9} y^{11} + x^{10} y^{11} \\ \end{aligned}$$

Hence the result.

#### **Theorem 2**

*The topological indices based on degree and neighbourhood degree sum for the isomorphic molecular graph* $P\left( {V,E} \right)$ *of Posaconazole are*.

1. $$M_{1} \left( P \right) = 422, NM_{1} \left( P \right) = 993$$
2. $$M_{2} \left( P \right) = 630, NM_{2} \left( P \right) = 3540$$
3. $$mM_{2} \left( P \right) = 30.0833, \;NmM_{2} \left( P \right) = 1.8183$$
4. $${\text{ReZ}}G_{3} \left( P \right) = 3984, ND_{3} \left( P \right) = 53700$$
5. $$F\left( P \right) = 1332, NF\left( P \right) = 7425$$
6. $$SDD\left( P \right) = 154.1665, ND_{5} \left( P \right) = 150.5053$$
7. $$H\left( P \right) = 24.9119, NH\left( P \right) = 10.723$$
8. $$I\left( P \right) = 102.2047, NI\left( P \right) = 241.978$$

#### Proof

 Let, $$M\left( {P;x,y} \right) = f\left( {x,y} \right) = 2xy^{2} + 2xy^{3} + xy^{4} + 4x^{2} y^{2} + 10x^{2} y^{3} + 6x^{2} y^{4} + 24x^{3} y^{3} + 19x^{3} y^{4} + 3x^{4} y^{4}$$.

Then,

$$\begin{aligned} \left( {D_{x} + D_{y} } \right)\left( {f\left( {x,y} \right)} \right) & = 6xy^{2} + 8xy^{3} + 5xy^{4} + 16x^{2} y^{2} + 50x^{2} y^{3} + 36x^{2} y^{4} \\ & \quad + 144x^{3} y^{3} + 133x^{3} y^{4} + 24x^{4} y^{4} \\ \end{aligned}$$

$$\begin{aligned} \left( {D_{x} D_{y} } \right)\left( {f\left( {x,y} \right)} \right) & = 4xy^{2} + 6xy^{3} + 4xy^{4} + 16x^{2} y^{2} + 60x^{2} y^{3} + 48x^{2} y^{4} + 216x^{3} y^{3} \\ & \quad + 228x^{3} y^{4} + 4x^{4} y^{4} \\ \end{aligned}$$

$$\begin{aligned} \left( {D_{x}^{2} + D_{y}^{2} } \right)\left( {f\left( {x,y} \right)} \right) & = 10xy^{2} + 20xy^{3} + 17xy^{4} + 32x^{2} y^{2} + 130x^{2} y^{3} + 120x^{2} y^{4} \\ & \quad + 432x^{3} y^{3} + 475x^{3} y^{4} + 96x^{4} y^{4} \\ \end{aligned}$$

$$\begin{aligned} \left( {D_{x} D_{y} } \right){ }\left( {D_{x} + D_{y} } \right)\left( {f\left( {x,y} \right)} \right) & = 12xy^{2} + 24xy^{3} + 20xy^{4} + 64x^{2} y^{2} + 300x^{2} y^{3} \\ & \quad + 288x^{2} y^{4} + 1296x^{3} y^{3} + 1596x^{3} y^{4} + 384x^{4} y^{4} \\ \end{aligned}$$

$$\begin{aligned} \left( {I_{y} D_{x} + I_{x} D_{y} } \right)\left( {f\left( {x,y} \right)} \right) & = 5xy^{2} + 6.6666xy^{3} + 4.25xy^{4} + 8x^{2} y^{2} + 21.6666x^{2} y^{3} \\ & \quad + 15x^{2} y^{4} + 48x^{3} y^{3} + 39.5833x^{3} y^{4} + 6x^{4} y^{4} \\ \end{aligned}$$

$$\begin{aligned} \left( {2I_{x} J} \right)\left( {f\left( {x,y} \right)} \right) & = 1.3333xy^{2} + xy^{3} + 0.4xy^{4} + 2x^{2} y^{2} + 4x^{2} y^{3} + 2x^{2} y^{4} + 8x^{3} y^{3} \\ & \quad + 5.4285x^{3} y^{4} + 0.75x^{4} y^{4} \\ \end{aligned}$$

$$\begin{aligned} \left( {I_{x} JD_{x} D_{y} } \right)\left( {f\left( {x,y} \right)} \right) & = 1.3333xy^{2} + 1.5xy^{3} + 0.8xy^{4} + 4x^{2} y^{2} + 12x^{2} y^{3} + 8x^{2} y^{4} \\ & \quad + 36x^{3} y^{3} + 32.5714x^{3} y^{4} + 6x^{4} y^{4} \\ \end{aligned}$$

Now from Table 1,

1. $$M_{1} \left( P \right) = \left( {D_{x} + D_{y} } \right)\left( {f\left( {x,y} \right)} \right)|_{x = y = 1} = 422$$
2. $$M_{2} \left( P \right) = \left( {D_{x} D_{y} } \right)\left( {f\left( {x,y} \right)} \right)|_{x = y = 1} = 630$$
3. $$mM_{2} \left( P \right) = \left( {I_{x} I_{y} } \right)\left( {f\left( {x,y} \right)} \right)|_{x = y = 1} = 30.0833$$
4. $${\text{ReZ}}G_{3} \left( P \right) = \left( {D_{x} D_{y} } \right){ }\left( {D_{x} + D_{y} } \right)\left( {f\left( {x,y} \right)} \right)|_{x = y = 1} = 3984$$
5. $$F\left( P \right) = \left( {D_{x}^{2} + D_{y}^{2} } \right)\left( {f\left( {x,y} \right)} \right)|_{x = y = 1} = 1332$$
6. $$SDD\left( P \right) = \left( {I_{y} D_{x} + I_{x} D_{y} } \right)\left( {f\left( {x,y} \right)} \right)|_{x = y = 1} = 154.1665$$
7. $$H\left( P \right) = \left( {2I_{x} J} \right)\left( {f\left( {x,y} \right)} \right)|_{x = 1} = 24.9119$$
8. $$I\left( P \right) = \left( {I_{x} JD_{x} D_{y} } \right)\left( {f\left( {x,y} \right)} \right)|_{x = 1} = 102.2047$$

Now consider the $NM$-Polynomial

$$\begin{aligned} NM\left( {P;x,y} \right) & = f\left( {x,y} \right) = 2x^{2} y^{4} + 2x^{3} y^{6} + x^{4} y^{6} + 4x^{4} y^{8} + 2x^{5} y^{5} \\ & \quad + 4x^{5} y^{6} + 4x^{5} y^{8} + 8x^{6} y^{6} + 4x^{6} y^{7} + 6x^{6} y^{8} + 2x^{6} y^{9} + 2x^{6} y^{10} + x^{6} y^{11} \\ & \quad + 5x^{7} y^{7} + 4x^{7} y^{8} + 7x^{7} y^{9} + 2x^{7} y^{10} + x^{7} y^{11} + x^{8} y^{8} + 2x^{8} y^{9} + 2x^{8} y^{10} \\ & \quad + 3x^{8} y^{11} + x^{9} y^{11} + x^{10} y^{11} \\ \end{aligned}$$

In similar manner the topological indices based on neighbourhood degree sum are deduced from the above polynomial.

#### **Theorem 3**

*Let* $I\left( {V,E} \right)$ *be the isomorphic molecular graph of Isavuconazole, then the* $M$ *and* $NM$ *polynomials of* $I$ *are*.

$$M\left( {I;x,y} \right) = xy^{2} + xy^{3} + 2x^{ } y^{4} + 2x^{2} y^{3} + 3x^{2} y^{4} + 12x^{3} y^{3} + 21x^{3} y^{4} + 5x^{4} y^{4}$$

$$\begin{aligned} NM\left( {I;x,y} \right) & = x^{2} y^{5} + x^{3} y^{9} + 4x^{4} y^{7} + x^{4} y^{8} + x^{5} y^{11} + 5x^{6} y^{6} + x^{6} y^{7} \\ & \quad + 2x^{6} y^{8} + 2x^{6} y^{10} + 6x^{7} y^{7} + 5x^{7} y^{8} + 7x^{7} y^{10} + x^{7} y^{11} + 2x^{8} y^{8} + x^{8} y^{9} \\ & \quad + x^{8} y^{10} + 3x^{8} y^{11} + x^{9} y^{11} + x^{10} y^{10} + x^{11} y^{11} \\ \end{aligned}$$

#### Proof

**Fig. 4:**
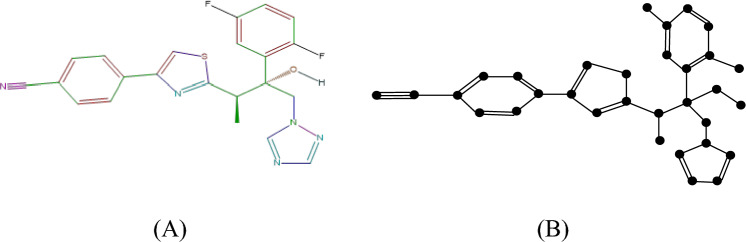
(**A**) Chemical structure and (**B**) Isomorphic molecular graph of Isavuconazole.

 The molecular structure and isomorphic molecular graph representation of Isavuconazole are depicted in Fig. 4A,B respectively. Let $I$ be the isomorphic molecular graph of Isavuconazole with 32 vertices (atoms) and 47 edges(bonds). Let $E_{i,j} = \left\{ {uv \in E\left( G \right):d_{u} = i, d_{v} = j} \right\}$ and $\left| {E_{i,j} } \right| =$ $e_{i,j} .$ From the isomorphic molecular graph the degree-based edge partitions are

$$e_{1,2} = 1, e_{1,3} = 1,e_{2,3} = 2, e_{2,4} = 3, e_{3,3} = 12, e_{3,4} = 21, e_{4,4} = 5,e_{1,4} = 2.$$

From the definition of $M$-Polynomial,

$$\begin{aligned} M\left( {I;x,y} \right) & = \mathop \sum \limits_{\delta \le i \le j \le \Delta } e_{i,j} x^{i} y^{j} \\ & = e_{1,2} xy^{2} + e_{1,3} xy^{3} + e_{1,4} x^{ } y^{4} + { }e_{2,3} x^{2} y^{3} + e_{2,4} x^{2} y^{4} + e_{3,3} x^{3} y^{3} + e_{3,4} x^{3} y^{4} + e_{4,4} x^{4} y^{4} \\ & = xy^{2} + xy^{3} + 2x^{ } y^{4} + 2x^{2} y^{3} + 3x^{2} y^{4} + 12x^{3} y^{3} + 21x^{3} y^{4} + 5x^{4} y^{4} \\ \end{aligned}$$

Similarly, let $E_{i,j}^{*} = \left\{ {uv \in E\left( G \right):nd_{u} = i, nd_{v} = j} \right\}$ and $\left| {E_{i,j} } \right| =$ $e_{i,j}^{*}$. Thus, from Fig. 4B, the neighbourhood degree sum-based edge partitions are

$$\begin{gathered} e_{2,5}^{*} = 1,e_{3,9}^{*} = 1,e_{4,7}^{*} = 4,e_{4,8}^{*} = 1,e_{5,11}^{*} = 1,e_{6,6}^{*} = 5,e_{6,7}^{*} = 1,e_{6,8}^{*} = 2,e_{6,10}^{*} = 2,e_{7,7}^{*} = 6, \hfill \\ e_{7,8}^{*} = 5,e_{7,10}^{*} = 7,e_{8,8}^{*} = 2,e_{8,9}^{*} = 1,e_{8,10}^{*} = 1,e_{8,11}^{*} = 3,e_{9,11}^{*} = 1,e_{7,11}^{*} = 1, e_{10,10}^{*} = 1,e_{11,11}^{*} = 1. \hfill \\ \end{gathered}$$

Now from the definition of $NM$-Polynomial,

$$\begin{aligned} NM\left( {I;x,y} \right) & = \mathop \sum \limits_{\delta \le i \le j \le \Delta } e_{i,j}^{*} x^{i} y^{j} . \\ & = e_{2,5}^{*} x^{2} y^{5} + e_{3,9}^{*} x^{3} y^{9} + e_{4,7}^{*} x^{4} y^{7} + e_{4,8}^{*} x^{4} y^{8} + e_{5,11}^{*} x^{5} y^{11} \\ & \quad + e_{6,6}^{*} x^{6} y^{6} + e_{6,7}^{*} x^{6} y^{7} + e_{6,8}^{*} x^{6} y^{8} + e_{6,10}^{*} x^{6} y^{10} + e_{7,7}^{*} x^{7} y^{7} + e_{7,8}^{*} x^{7} y^{8} \\ & \quad + e_{7,10}^{*} x^{7} y^{10} + e_{8,8}^{*} x^{8} y^{8} + e_{8,9}^{*} x^{8} y^{9} + e_{8,10}^{*} x^{8} y^{10} + e_{8,11}^{*} x^{8} y^{11} + e_{9,11}^{*} x^{9} y^{11} \\ & \quad + e_{7,11}^{*} x^{7} y^{11} + e_{10,10}^{*} x^{10} y^{10} + e_{11,11}^{*} x^{11} y^{11} . \\ & = x^{2} y^{5} + x^{3} y^{9} + 4x^{4} y^{7} + x^{4} y^{8} + x^{5} y^{11} + 5x^{6} y^{6} + x^{6} y^{7} \\ & \quad 2x^{6} y^{8} + 2x^{6} y^{10} + 6x^{7} y^{7} + 5x^{7} y^{8} + 7x^{7} y^{10} + 2x^{8} y^{8} + x^{8} y^{9} + x^{8} y^{10} \\ & \quad + 3x^{8} y^{11} + x^{9} y^{11} + x^{7} y^{11} + x^{10} y^{10} + x^{11} y^{11} . \\ \end{aligned}$$

Hence the theorem.

#### **Theorem 4**

*Let* $I\left( {V,E} \right)$ *be the isomorphic molecular graph of Isavuconazole, then*.

1. $$M_{1} \left( I \right) = 304, NM_{1} \left( I \right) = 706$$
2. $$M_{2} \left( I \right) = 489, NM_{2} \left( I \right) = 2679$$
3. $$mM_{2} \left( I \right) = 5.4375, NmM_{2} \left( I \right) = 1.0323$$
4. $${\text{ReZ}}G_{3} \left( I \right) = 3314,ND_{3} \left( I \right) = 43262$$
5. $$F\left( I \right) = 1036,NF\left( I \right) = 5652$$
6. $$SDD\left( I \right) = 103.9166, ND_{5} \left( I \right) = 101.0055$$
7. $$H\left( I \right) = 15.0166,NH\left( I \right) = 6.5381$$
8. $$I\left( I \right) = 73.4166,NI\left( I \right) = 171.3449$$

#### **Theorem 5**

* Let* $A\left( {V,E} \right)$ *be the isomorphic molecular graph of Amphotericin B, then the* $M$ *and* $NM$ *polynomials of the molecular graph* $A$ *are*

$$M\left( {A;x,y} \right) = 11xy^{2} + 6xy^{3} + x^{2} y^{2} + 26x^{2} y^{3} + 11x^{2} y^{4} + 33x^{3} y^{3} + x^{3} y^{4}$$

$$\begin{aligned} NM\left( {A;x,y} \right) & = 9x^{2} y^{4} + 2x^{2} y^{5} + 2x^{3} y^{5} + 2x^{3} y^{6} + 2x^{3} y^{7} + 5x^{4} y^{6} \\ & \quad + 5x^{4} y^{7} + 3x^{4} y^{8} + x^{5} y^{5} + x^{5} y^{6} + 2x^{5} y^{7} + x^{5} y^{8} + x^{5} y^{9} + 24x^{6} y^{6} \\ & \quad + 14x^{6} y^{7} + x^{6} y^{8} + 3x^{7} y^{7} + 6x^{7} y^{8} + 3x^{7} y^{10} + 2x^{8} y^{9} . \\ \end{aligned}$$

#### Proof

**Fig. 5:**
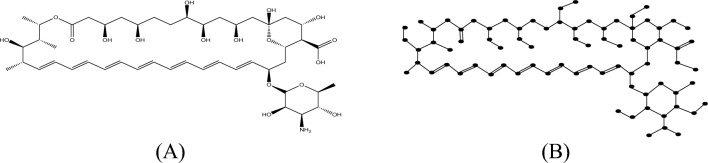
(**A**) Chemical structure and (**B**) Isomorphic molecular graph of Amphotericin B.

 The molecular structure and isomorphic graph representation of Amphotericin B are depicted in Fig. 5A,B respectively. Let $A$ be the isomorphic molecular graph of Amphotericin B with 78 vertices (atoms) and 89 edges(bonds). Let $E_{i,j} = \left\{ {uv \in E\left( G \right):d_{u} = i, d_{v} = j} \right\}$ and $\left| {E_{i,j} } \right| =$ $e_{i,j} .$ From Fig. 5B, the degree-based edge partitions are

$$e_{1,2} = 11,e_{1,3} = 6, e_{2,2} = 1, e_{2,3} = 26, e_{2,4} = 11, e_{3,3} = 33,e_{3,4} = 1.$$

From the definition of $M$-Polynomial

$$\begin{aligned} M\left( {A;x,y} \right) & = \mathop \sum \limits_{\delta \le i \le j \le \Delta } e_{i,j} x^{i} y^{j} \\ & = e_{1,2} xy^{2} + e_{1,3} xy^{3} + e_{2,2} x^{2} y^{2} + e_{2,3} x^{2} y^{3} + e_{2,4} x^{2} y^{4} + e_{3,3} x^{3} y^{3} + e_{3,4} x^{3} y^{4} . \\ & = 11xy^{2} + 6xy^{3} + x^{2} y^{2} + 26x^{2} y^{3} + 11x^{2} y^{4} + 33x^{3} y^{3} + x^{3} y^{4} \\ \end{aligned}$$

Similarly, let $E_{i,j}^{*} = \left\{ {uv \in E\left( G \right):nd_{u} = i, nd_{v} = j} \right\}$ and $\left| {E_{i,j} } \right| =$ $e_{i,j}^{*}$. Thus, from Fig. 5B, the neighbourhood degree sum-based edge partitions are

$$\begin{gathered} e_{2,4}^{*} = 9,e_{2,5}^{*} = 2,e_{3,5}^{*} = 2,e_{3,6}^{*} = 2,e_{3,7}^{*} = 2,e_{4,6}^{*} = 5,e_{4,7}^{*} = 5,e_{4,8}^{*} = 3,e_{5,5}^{*} = 1, \hfill \\ e_{5,6}^{*} = 1,e_{5,7}^{*} = 2,e_{5,8}^{*} = 1,e_{5,9}^{*} = 1,e_{6,6}^{*} = 24, \hfill \\ e_{6,7}^{*} = 14,e_{6,8}^{*} = 1,e_{7,7}^{*} = 3,e_{7,8}^{*} = 6,e_{7,10}^{*} = 3, e_{8,9}^{*} = 2. \hfill \\ \end{gathered}$$

Now from the definition of $NM$-Polynomial,

$$\begin{aligned} NM\left( {A;x,y} \right) & = \mathop \sum \limits_{\delta \le i \le j \le \Delta } e_{i,j}^{*} x^{i} y^{j} \\ & = e_{2,4}^{*} x^{2} y^{4} + e_{2,5}^{*} x^{2} y^{5} + e_{3,5}^{*} x^{3} y^{5} + e_{3,6}^{*} x^{3} y^{6} + e_{3,7}^{*} x^{3} y^{7} \\ & \quad + e_{4,6}^{*} x^{4} y^{6} + e_{4,7}^{*} x^{4} y^{7} + e_{4,8}^{*} x^{4} y^{8} + e_{5,5}^{*} x^{5} y^{5} + e_{5,6}^{*} x^{5} y^{6} + e_{5,7}^{*} x^{5} y^{7} \\ & \quad + e_{5,8}^{*} x^{5} y^{8} + e_{5,9}^{*} x^{5} y^{9} + e_{6,6}^{*} x^{6} y^{6} + e_{6,7}^{*} x^{6} y^{7} + e_{6,8}^{*} x^{6} y^{8} + e_{7,7}^{*} x^{7} y^{7} \\ & \quad + e_{7,8}^{*} x^{7} y^{8} + e_{7,10}^{*} x^{7} y^{10} + e_{8,9}^{*} x^{8} y^{9} . \\ & = 9x^{2} y^{4} + 2x^{2} y^{5} + 2x^{3} y^{5} + 2x^{3} y^{6} + 2x^{3} y^{7} + 5x^{4} y^{6} \\ & \quad + 5x^{4} y^{7} + 3x^{4} y^{8} + x^{5} y^{5} + x^{5} y^{6} + 2x^{5} y^{7} + x^{5} y^{8} + x^{5} y^{9} + 24x^{6} y^{6} \\ & \quad + 14x^{6} y^{7} + x^{6} y^{8} + 3x^{7} y^{7} + 6x^{7} y^{8} + 3x^{7} y^{10} + 2x^{8} y^{9} . \\ \end{aligned}$$

Hence the result.

#### **Theorem 6**

* Let* $A\left( {V,E} \right)$ *be the isomorphic molecular graph of Amphotericin B, then*.

1. $$M_{1} \left( A \right) = 462, NM_{1} \left( A \right) = {1}0{36}$$
2. $$M_{2} \left( A \right) = 597, NM_{2} \left( A \right) = {31}0{3}$$
3. $$mM_{2} \left( A \right) = 17.2083, NmM_{2} \left( A \right) = 3.5828$$
4. $${\text{ReZ}}G_{3} \left( A \right) = 3328,ND_{3} \left( A \right) = 39818$$
5. $$F\left( A \right) = 1300,NF\left( A \right) = {6518}$$
6. $$SDD\left( A \right) = 201.4166, ND_{5} \left( A \right) = {193}.0{773}$$
7. $$H\left( A \right) = 30.6857,NH\left( A \right) = {16}.{45}0{3}$$
8. $$I\left( A \right) = 109.9142,NI\left( A \right) = {251}.{2691}$$

#### **Theorem 7**

*Let* $A_{1} \left( {V,E} \right)$ *be the isomorphic molecular graph of Analog* $A_{1}$ *of the drug Amphotericin B, then the* $M$ *and* $NM$ *polynomials of* $A_{1}$ *are*

$$M\left( {A_{1} ;x,y} \right) = 10xy^{2} + 7xy^{3} + x^{2} y^{2} + 26x^{2} y^{3} + 12x^{2} y^{4} + 38x^{3} y^{3} + 8x^{3} y^{4} + 2x^{4} y^{4} .$$

$$\begin{aligned} NM\left( {A_{1} ;x,y} \right) & = 9x^{2} y^{4} + x^{2} y^{5} + 2x^{3} y^{5} + 2x^{3} y^{6} + 2x^{3} y^{7} + x^{3} y^{9} + 5x^{4} y^{6} \\ & \quad + 5x^{4} y^{7} + 3x^{4} y^{8} + x^{5} y^{5} + x^{5} y^{6} + x^{5} y^{7} + x^{5} y^{8} + x^{5} y^{9} \\ & \quad + 25x^{6} y^{6} + 18x^{6} y^{7} + x^{6} y^{8} + 3x^{7} y^{7} + 7x^{7} y^{8} + 5x^{7} y^{10} \\ & \quad + 3x^{8} y^{8} + 3x^{8} y^{9} + x^{8} y^{10} + x^{9} y^{10} + 2x^{10} y^{10} . \\ \end{aligned}$$

#### Proof

**Fig. 6:**
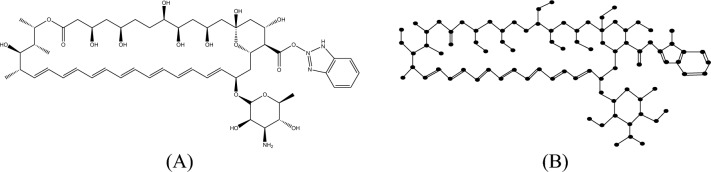
(**A**) Chemical structure and (**B**) Isomorphic molecular graph of Analog $A_{1}$.

The molecular structure and isomorphic molecular graph representation of Analog $A_{1}$ are depicted in Fig. 6A,B respectively. Let $A_{1}$ be the isomorphic molecular graph of Analog $A_{1}$ with 87 vertices (atoms) and 104 edges (bonds). Let $E_{i,j} = \left\{ {uv \in E\left( G \right):d_{u} = i, d_{v} = j} \right\}$ and $\left| {E_{i,j} } \right| =$ $e_{i,j} .$ From Fig. 6B, the degree-based edge partitions are

$$e_{1,2} = 11,e_{1,3} = 6, e_{2,2} = 1, e_{2,3} = 26, e_{2,4} = 11, e_{3,3} = 33,e_{3,4} = 1.$$

From the definition of $M$-Polynomial

$$\begin{aligned} M\left( {A_{1} ;x,y} \right) & = \mathop \sum \limits_{\delta \le i \le j \le \Delta } e_{i,j} x^{i} y^{j} \\ & = e_{1,2} xy^{2} + e_{1,3} xy^{3} + e_{2,2} x^{2} y^{2} + e_{2,3} x^{2} y^{3} + e_{2,4} x^{2} y^{4} + e_{3,3} x^{3} y^{3} \\ & \quad + e_{3,4} x^{3} y^{4} + e_{4,4} x^{4} y^{4} . \\ & = 10xy^{2} + 7xy^{3} + x^{2} y^{2} + 26x^{2} y^{3} + 12x^{2} y^{4} + 38x^{3} y^{3} + 8x^{3} y^{4} + 2x^{4} y^{4} . \\ \end{aligned}$$

Similarly, let $E_{i,j}^{*} = \left\{ {uv \in E\left( G \right):nd_{u} = i, nd_{v} = j} \right\}$ and $\left| {E_{i,j} } \right| =$ $e_{i,j}^{*}$. Thus, from Fig. 6B, the neighbourhood degree sum-based edge partitions are

$$\begin{gathered} e_{2,4}^{*} = 9,e_{2,5}^{*} = 2,e_{3,5}^{*} = 2,e_{3,6}^{*} = 2,e_{3,7}^{*} = 2,e_{4,6}^{*} = 5,e_{4,7}^{*} = 5,e_{4,8}^{*} = 3,e_{5,5}^{*} = 1, \hfill \\ e_{5,6}^{*} = 1,e_{5,7}^{*} = 2,e_{5,8}^{*} = 1,e_{5,9}^{*} = 1,e_{6,6}^{*} = 24,e_{6,7}^{*} = 14,e_{6,8}^{*} = 1,e_{7,7}^{*} = 3,e_{7,8}^{*} = 6, \hfill \\ e_{7,10}^{*} = 3, e_{8,8}^{*} = 3,e_{8,9}^{*} = 2,e_{8,9}^{*} = 3,e_{9,10}^{*} = 1,e_{10,10}^{*} = 2. \hfill \\ \end{gathered}$$

Now from the definition of $NM$-Polynomial,

$$\begin{aligned} NM\left( {A_{1} ;x,y} \right) & = \mathop \sum \limits_{\delta \le i \le j \le \Delta } e_{i,j}^{*} x^{i} y^{j} \\ & = e_{2,4}^{*} x^{2} y^{4} + e_{2,5}^{*} x^{2} y^{5} + e_{3,5}^{*} x^{3} y^{5} + e_{3,6}^{*} x^{3} y^{6} + e_{3,7}^{*} x^{3} y^{7} \\ & \quad + e_{3,9}^{*} x^{3} y^{9} + e_{4,6}^{*} x^{4} y^{6} + e_{4,7}^{*} x^{4} y^{7} + e_{4,8}^{*} x^{4} y^{8} + e_{5,5}^{*} x^{5} y^{5} + e_{5,6}^{*} x^{5} y^{6} \\ & \quad + e_{5,7}^{*} x^{5} y^{7} + e_{5,8}^{*} x^{5} y^{8} + e_{5,9}^{*} x^{5} y^{9} + e_{6,6}^{*} x^{6} y^{6} + e_{6,7}^{*} x^{6} y^{7} + e_{6,8}^{*} x^{6} y^{8} \\ & \quad + e_{7,7}^{*} x^{7} y^{7} + e_{7,8}^{*} x^{7} y^{8} + e_{7,10}^{*} x^{7} y^{10} + e_{8,8}^{*} x^{8} y^{8} + e_{8,9}^{*} x^{8} y^{9} + e_{8,10}^{*} x^{8} y^{10} \\ & \quad + e_{9,10}^{*} x^{9} y^{10} + e_{10,10}^{*} x^{10} y^{10} . \\ & = 9x^{2} y^{4} + x^{2} y^{5} + 2x^{3} y^{5} + 2x^{3} y^{6} + 2x^{3} y^{7} + x^{3} y^{9} + 5x^{4} y^{6} \\ & \quad + 5x^{4} y^{7} + 3x^{4} y^{8} + x^{5} y^{5} + x^{5} y^{6} + x^{5} y^{7} + x^{5} y^{8} + x^{5} y^{9} + 25x^{6} y^{6} \\ & \quad + 18x^{6} y^{7} + x^{6} y^{8} + 3x^{7} y^{7} + 7x^{7} y^{8} + 5x^{7} y^{10} + 3x^{8} y^{8} + 3x^{8} y^{9} + x^{8} y^{10} \\ & \quad + x^{9} y^{10} + 2x^{10} y^{10} . \\ \end{aligned}$$

Hence the proof completed.

#### **Theorem 8**

* Let* $A_{1} \left( {V,E} \right)$ *be the isomorphic molecular graph of Analog* $A_{1}$, *then*.

1. $$M_{1} \left( {A_{1} } \right) = 564, NM_{1} \left( {A_{1} } \right) =$$ 1284
2. $$M_{2} \left( {A_{1} } \right) = 767, NM_{2} \left( {A_{1} } \right) =$$ 4119
3. $$mM_{2} \left( {A_{1} } \right) = 18.4305, NmM_{2} \left( {A_{1} } \right) = 3.7678$$
4. $${\text{ReZ}}G_{3} \left( {A_{1} } \right) = 4496,ND_{3} \left( {A_{1} } \right) = 56934$$
5. $$F\left( {A_{1} } \right) = 1654,NF\left( {A_{1} } \right) = {86}0{2}$$
6. $$SDD\left( {A_{1} } \right) = 233.3332, ND_{5} \left( {A_{1} } \right) = {223}.{8416}$$
7. $$H\left( {A_{1} } \right) = 40.5190,NH\left( {A_{1} } \right) = {18}.{3386}$$
8. $$I\left( {A_{1} } \right) = 134.8309,NI\left( {A_{1} } \right) = {313}.0{535}$$

#### **Theorem 9**

*Let* $A_{2} \left( {V,E} \right)$ *be the isomorphic molecular graph of Analog* $A_{2}$ *of the drug Amphotericin B, then the* $M$ *and* $NM$ *polynomials of* $A_{2}$ *are*

$$M\left( {A_{2} ;x,y} \right) = 10xy^{2} + 7xy^{3} + x^{2} y^{2} + 28x^{2} y^{3} + 12x^{2} y^{4} + 39x^{3} y^{3} + 4x^{3} y^{4}$$

$$\begin{aligned} NM\left( {A_{2} ;x,y} \right) & = 9x^{2} y^{4} + x^{2} y^{5} + 3x^{3} y^{5} + 2x^{3} y^{6} + 2x^{3} y^{7} + 5x^{4} y^{6} \\ & \quad + 5x^{4} y^{7} + 3x^{4} y^{8} + x^{5} y^{5} + x^{5} y^{6} + 3x^{5} y^{7} + x^{5} y^{8} + x^{5} y^{9} + 27x^{6} y^{6} \\ & \quad + 17x^{6} y^{7} + x^{6} y^{8} + 4x^{7} y^{7} + 10x^{7} y^{8} + 3x^{7} y^{10} + 2x^{8} y^{9} . \\ \end{aligned}$$

#### Proof

**Fig. 7:**
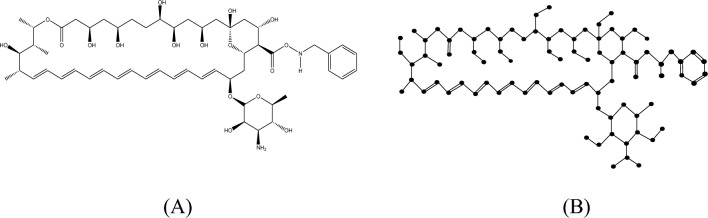
(**A**) Chemical structure and (**B**) Isomorphic molecular graph of Analog $A_{2} .$

The molecular structure and isomorphic molecular graph representation of Analog $A_{2}$ are depicted in Fig. 7A,B respectively. Let $A_{2}$ be the molecular graph of Analog 2 with 86 vertices (atoms) and 101 edges (bonds). Let $E_{i,j} = \left\{ {uv \in E\left( G \right):d_{u} = i, d_{v} = j} \right\}$ and $\left| {E_{i,j} } \right| =$ $e_{i,j} .$ From Fig. 7B, the degree-based edge partitions are $e_{1,2} = 10,e_{1,3} = 7, e_{2,2} = 1, e_{2,3} = 28, e_{2,4} = 12, e_{3,3} = 39,e_{3,4} = 4.$

From the definition of $M$-Polynomial

$$\begin{aligned} M\left( {A_{2} ;x,y} \right) & = \mathop \sum \limits_{\delta \le i \le j \le \Delta } e_{i,j} x^{i} y^{j} \\ & = e_{1,2} xy^{2} + e_{1,3} xy^{3} + e_{2,2} x^{2} y^{2} + e_{2,3} x^{2} y^{3} + e_{2,4} x^{2} y^{4} + e_{3,3} x^{3} y^{3} + e_{3,4} x^{3} y^{4} . \\ & = 10xy^{2} + 7xy^{3} + x^{2} y^{2} + 28x^{2} y^{3} + 12x^{2} y^{4} + 39x^{3} y^{3} + 4x^{3} y^{4} \\ \end{aligned}$$

Similarly, let $E_{i,j}^{*} = \left\{ {uv \in E\left( G \right):nd_{u} = i, nd_{v} = j} \right\}$ and $\left| {E_{i,j} } \right| =$ $e_{i,j}^{*}$. Thus, from Fig. 7B, the neighbourhood degree sum-based edge partitions are

$$\begin{gathered} e_{2,4}^{*} = 9,e_{2,5}^{*} = 1,e_{3,5}^{*} = 3,e_{3,6}^{*} = 2,e_{3,7}^{*} = 2,e_{4,6}^{*} = 5,e_{4,7}^{*} = 5,e_{4,8}^{*} = 3,e_{5,5}^{*} = 1, \hfill \\ e_{5,6}^{*} = 1,e_{5,7}^{*} = 3,e_{5,8}^{*} = 1,e_{5,9}^{*} = 1,e_{6,6}^{*} = 27,e_{6,7}^{*} = 17,e_{6,8}^{*} = 1,e_{7,7}^{*} = 4,e_{7,8}^{*} = \hfill \\ 10,e_{7,10}^{*} = 3, e_{8,9}^{*} = 2. \hfill \\ \end{gathered}$$

Therefore, from the definition of $NM$-Polynomial,

$$\begin{aligned} NM\left( {A_{2} ;x,y} \right) & = \mathop \sum \limits_{\delta \le i \le j \le \Delta } e_{i,j}^{*} x^{i} y^{j} \\ & = e_{2,4}^{*} x^{2} y^{4} + e_{2,5}^{*} x^{2} y^{5} + e_{3,5}^{*} x^{3} y^{5} + e_{3,6}^{*} x^{3} y^{6} + e_{3,7}^{*} x^{3} y^{7} \\ & \quad + e_{4,6}^{*} x^{4} y^{6} + e_{4,7}^{*} x^{4} y^{7} + e_{4,8}^{*} x^{4} y^{8} + e_{5,5}^{*} x^{5} y^{5} + e_{5,6}^{*} x^{5} y^{6} + e_{5,7}^{*} x^{5} y^{7} \\ & \quad + e_{5,8}^{*} x^{5} y^{8} + e_{5,9}^{*} x^{5} y^{9} + e_{6,6}^{*} x^{6} y^{6} + e_{6,7}^{*} x^{6} y^{7} + e_{6,8}^{*} x^{6} y^{8} + e_{7,7}^{*} x^{7} y^{7} \\ & \quad + e_{7,8}^{*} x^{7} y^{8} + e_{7,10}^{*} x^{7} y^{10} + e_{8,9}^{*} x^{8} y^{9} . \\ & = 9x^{2} y^{4} + x^{2} y^{5} + 3x^{3} y^{5} + 2x^{3} y^{6} + 2x^{3} y^{7} + 5x^{4} y^{6} \\ & \quad + 5x^{4} y^{7} + 3x^{4} y^{8} + x^{5} y^{5} + x^{5} y^{6} + 3x^{5} y^{7} + x^{5} y^{8} + x^{5} y^{9} + 27x^{6} y^{6} \\ & \quad + 17x^{6} y^{7} + x^{6} y^{8} + 4x^{7} y^{7} + 10x^{7} y^{8} + 3x^{7} y^{10} + 2x^{8} y^{9} . \\ \end{aligned}$$

This completes the proof.

#### **Theorem 10**

*Let* $A_{2} \left( {V,E} \right)$ *be the isomorphic molecular graph of Analog* $A_{2}$, *then*

1. $$M_{1} \left( {A_{2} } \right) = 536, NM_{1} \left( {A_{2} } \right) = {1198}$$
2. $$M_{2} \left( {A_{2} } \right) = 708, NM_{2} \left( {A_{2} } \right) = {365}0$$
3. $$mM_{2} \left( {A_{2} } \right) = 18.4166, NmM_{2} \left( {A_{2} } \right) = 3.8024$$
4. $${\text{ReZ}}G_{3} \left( {A_{2} } \right) = 4018,ND_{3} \left( {A_{2} } \right) = 47268$$
5. $$F\left( A \right) = 1534,NF\left( {A_{2} } \right) = {7618}$$
6. $$SDD\left( {A_{2} } \right) = 227.3333, ND_{5} \left( {A_{2} } \right) = {216}.{7}0{11}$$
7. $$H\left( {A_{2} } \right) = 40.0095,NH\left( {A_{2} } \right) = {18}.{219}0$$
8. $$I\left( {A_{2} } \right) = 127.8738,NI\left( {A_{2} } \right) = {291}.{7578}$$

#### **Theorem 11**

* Let* $A_{3} \left( {V,E} \right)$ *be the isomorphic molecular graph of Analog* $A_{3}$ *of the drug Amphotericin B, then the* $M$ *and* $NM$ *polynomials of* $A_{3}$ *are*

$$M\left( {A_{3} ;x,y} \right) = 10xy^{2} + 7xy^{3} + 5x^{2} y^{2} + 29x^{2} y^{3} + 11x^{2} y^{4} + 34x^{3} y^{3} + x^{3} y^{4}$$

$$\begin{aligned} NM\left( {A_{3} ;x,y} \right) & = 9x^{2} y^{4} + x^{2} y^{5} + 2x^{3} y^{5} + 3x^{3} y^{6} + 2x^{3} y^{7} + 2x^{4} y^{4} \\ & \quad + 2x^{4} y^{5} + 5x^{4} y^{6} + 5x^{4} y^{7} + 3x^{4} y^{8} + x^{5} y^{5} + x^{5} y^{6} + 3x^{5} y^{7} + x^{5} y^{8} \\ & \quad + x^{5} y^{9} + 24x^{6} y^{6} + 16x^{6} y^{7} + x^{6} y^{8} + 4x^{7} y^{7} + 6x^{7} y^{8} + 3x^{7} y^{10} + 2x^{8} y^{9} . \\ \end{aligned}$$

#### Proof

**Fig. 8:**
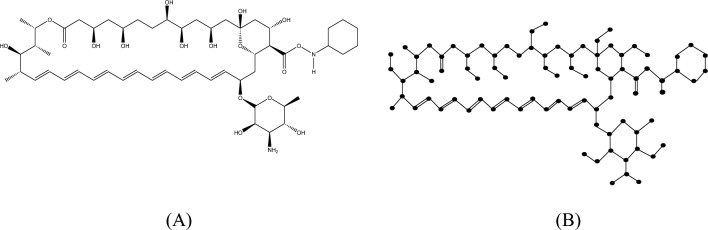
(**A**) Chemical structure and (**B**) Isomorphic molecular graph of Analog $A_{3}$.

The molecular structure and isomorphic molecular graph representation of Analog $A_{3}$ are depicted in Fig. 8A,B respectively. Let $A_{3}$ be the isomorphic molecular graph of Analog $A_{3}$ with 85 vertices (atoms) and 97 edges (bonds). Let $E_{i,j} = \left\{ {uv \in E\left( G \right):d_{u} = i, d_{v} = j} \right\}$ and $\left| {E_{i,j} } \right| =$ $e_{i,j} .$ From Fig. 8B, the degree-based edge partitions are $e_{1,2} = 10,e_{1,3} = 7, e_{2,2} = 5, e_{2,3} = 29, e_{2,4} = 11, e_{3,3} = 34,e_{3,4} = 1.$

From the definition of $M$-Polynomial

$$\begin{aligned} M\left( {A_{3} ;x,y} \right) & = \mathop \sum \limits_{\delta \le i \le j \le \Delta } e_{i,j} x^{i} y^{j} \\ & = e_{1,2} xy^{2} + e_{1,3} xy^{3} + e_{2,2} x^{2} y^{2} + e_{2,3} x^{2} y^{3} + e_{2,4} x^{2} y^{4} + e_{3,3} x^{3} y^{3} + e_{3,4} x^{3} y^{4} . \\ & = 10xy^{2} + 7xy^{3} + 5x^{2} y^{2} + 29x^{2} y^{3} + 11x^{2} y^{4} + 34x^{3} y^{3} + x^{3} y^{4} \\ \end{aligned}$$

Similarly, let $E_{i,j}^{*} = \left\{ {uv \in E\left( G \right):nd_{u} = i, nd_{v} = j} \right\}$ and $\left| {E_{i,j} } \right| =$ $e_{i,j}^{*}$. Thus, from Fig. 8B, the neighbourhood degree sum-based edge partitions are

$$\begin{gathered} e_{2,4}^{*} = 9,e_{2,5}^{*} = 1,e_{3,5}^{*} = 2,e_{3,6}^{*} = 3,e_{3,7}^{*} = 2,e_{4,4}^{*} = 2,e_{4,5}^{*} = 2,e_{4,6}^{*} = 5,e_{4,7}^{*} = 5,e_{4,8}^{*} = 3, \hfill \\ e_{5,5}^{*} = 1,e_{5,6}^{*} = 1,e_{5,7}^{*} = 3,e_{5,8}^{*} = 1,e_{5,9}^{*} = 1,e_{6,6}^{*} = 24,e_{6,7}^{*} = 16,e_{6,8}^{*} = 1,e_{7,7}^{*} = 4, \hfill \\ e_{7,8}^{*} = 6, e_{7,10}^{*} = 3, e_{8,9}^{*} = 2. \hfill \\ \end{gathered}$$

Therefore, from the definition of $NM$-Polynomial,

$$\begin{aligned} NM\left( {A_{3} ;x,y} \right) & = \mathop \sum \limits_{\delta \le i \le j \le \Delta } e_{i,j}^{*} x^{i} y^{j} \\ & = e_{2,4}^{*} x^{2} y^{4} + e_{2,5}^{*} x^{2} y^{5} + e_{3,5}^{*} x^{3} y^{5} + e_{3,6}^{*} x^{3} y^{6} + e_{3,7}^{*} x^{3} y^{7} + e_{4,4}^{*} x^{4} y^{4} \\ & \quad + e_{4,5}^{*} x^{4} y^{5} + e_{4,6}^{*} x^{4} y^{6} + e_{4,7}^{*} x^{4} y^{7} + e_{4,8}^{*} x^{4} y^{8} + e_{5,5}^{*} x^{5} y^{5} + e_{5,6}^{*} x^{5} y^{6} \\ & \quad + e_{5,7}^{*} x^{5} y^{7} + e_{5,8}^{*} x^{5} y^{8} + e_{5,9}^{*} x^{5} y^{9} + e_{6,6}^{*} x^{6} y^{6} + e_{6,7}^{*} x^{6} y^{7} + e_{6,8}^{*} x^{6} y^{8} \\ & \quad + e_{7,7}^{*} x^{7} y^{7} + e_{7,8}^{*} x^{7} y^{8} + e_{7,10}^{*} x^{7} y^{10} + e_{8,9}^{*} x^{8} y^{9} \\ & = 9x^{2} y^{4} + x^{2} y^{5} + 2x^{3} y^{5} + 3x^{3} y^{6} + 2x^{3} y^{7} + 2x^{4} y^{4} + 2x^{4} y^{5} \\ & \quad + 5x^{4} y^{6} + 5x^{4} y^{7} + 3x^{4} y^{8} + x^{5} y^{5} + x^{5} y^{6} + 3x^{5} y^{7} + x^{5} y^{8} + x^{5} y^{9} \\ & \quad + 24x^{6} y^{6} + 16x^{6} y^{7} + x^{6} y^{8} + 4x^{7} y^{7} + 6x^{7} y^{8} + 3x^{7} y^{10} + 2x^{8} y^{9} . \\ \end{aligned}$$

Hence the result.

#### **Theorem 12**

* Let* $A_{3} \left( {V,E} \right)$ *be the isomorphic molecular graph of Analog* $A_{3}$, *then*.

1. $$M_{1} \left( {A_{3} } \right) = 500, NM_{1} \left( {A_{3} } \right) = {1124}$$
2. $$M_{2} \left( {A_{3} } \right) = 641, NM_{2} \left( {A_{3} } \right) = {3351}$$
3. $$mM_{2} \left( {A_{3} } \right) = 18.6527, NmM_{2} \left( {A_{3} } \right) = 3.8600$$
4. $${\text{ReZ}}G_{3} \left( {A_{3} } \right) = 3542,ND_{3} \left( {A_{3} } \right) = 42724$$
5. $$F\left( {A_{3} } \right) = 1394,NF\left( {A_{3} } \right) = {7}0{22}$$
6. $$SDD\left( {A_{3} } \right) = 218.7499, ND_{5} \left( {A_{3} } \right) = {2}0{8}.{9392}$$
7. $$H\left( {A_{3} } \right) = 42.0523,NH\left( {A_{3} } \right) = {17}.{8627}$$
8. $$I\left( {A_{3} } \right) = 119.0976,NI\left( {A_{3} } \right) = {272}.{7346}$$

#### **Theorem 13**

*Let* $A_{4} \left( {V,E} \right)$ *be the isomorphic molecular graph of Analog* $A_{4}$ *of the drug Amphotericin B, then the* $M$ *and* $NM$ *polynomials of* $A_{4}$ *are*.

$$M\left( {A_{4} ;x,y} \right) = 10xy^{2} + 10xy^{3} + x^{2} y^{2} + 27x^{2} y^{3} + 11x^{2} y^{4} + 35x^{3} y^{3} + x^{3} y^{4}$$

$$\begin{aligned} NM\left( {A_{4} ;x,y} \right) & = 9x^{2} y^{4} + x^{2} y^{5} + 6x^{3} y^{5} + 2x^{3} y^{6} + 2x^{3} y^{7} + 5x^{4} y^{6} \\ & \quad + 5x^{4} y^{7} + 3x^{4} y^{8} + x^{5} y^{5} + x^{5} y^{6} + x^{5} y^{7} + 3x^{5} y^{8} + x^{5} y^{9} + 24x^{6} y^{6} \\ & \quad + 14x^{6} y^{7} + x^{6} y^{8} + 4x^{7} y^{7} + 7x^{7} y^{8} + 3x^{7} y^{10} + 2x^{8} y^{9} . \\ \end{aligned}$$

#### Proof

**Fig. 9:**
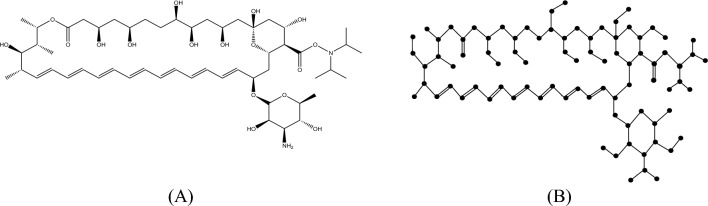
(**A**) Chemical structure and (**B**) Isomorphic molecular graph of Analog $A_{4}$.

The molecular structure and isomorphic molecular graph representation of Analog $A_{4}$ are depicted in Fig. 9A,B respectively. Let $A_{4}$ be the isomorphic molecular graph of Amphotericin B with 84 vertices (atoms) and 95 edges (bonds). Let $E_{i,j} = \left\{ {uv \in E\left( G \right):d_{u} = i, d_{v} = j} \right\}$ and $\left| {E_{i,j} } \right| =$ $e_{i,j} .$ From Fig. 9B, the degree-based edge partitions are $e_{1,2} = 10,e_{1,3} = 10, e_{2,2} = 1, e_{2,3} = 27, e_{2,4} = 11, e_{3,3} = 35,e_{3,4} = 1.$

From the definition of $M$-Polynomial

$$\begin{aligned} M\left( {A_{4} ;x,y} \right) & = \mathop \sum \limits_{\delta \le i \le j \le \Delta } e_{i,j} x^{i} y^{j} \\ & \quad = e_{1,2} xy^{2} + e_{1,3} xy^{3} + e_{2,2} x^{2} y^{2} + e_{2,3} x^{2} y^{3} + e_{2,4} x^{2} y^{4} + e_{3,3} x^{3} y^{3} + e_{3,4} x^{3} y^{4} . \\ & \quad = 10xy^{2} + 10xy^{3} + x^{2} y^{2} + 27x^{2} y^{3} + 11x^{2} y^{4} + 35x^{3} y^{3} + x^{3} y^{4} \\ \end{aligned}$$

Similarly, let $E_{i,j}^{*} = \left\{ {uv \in E\left( G \right):nd_{u} = i, nd_{v} = j} \right\}$ and $\left| {E_{i,j} } \right| =$ $e_{i,j}^{*}$. Thus, from Fig. 9B, the neighbourhood degree sum-based edge partitions are

$$\begin{gathered} e_{2,4}^{*} = 9,e_{2,5}^{*} = 1,e_{3,5}^{*} = 6,e_{3,6}^{*} = 2,e_{3,7}^{*} = 2,e_{4,6}^{*} = 5,e_{4,7}^{*} = 5,e_{4,8}^{*} = 3,e_{5,5}^{*} = 1,e_{5,6}^{*} = 1, \hfill \\ e_{5,7}^{*} = 1,e_{5,8}^{*} = 3,e_{5,9}^{*} = 1,e_{6,6}^{*} = 24,e_{6,7}^{*} = 14,e_{6,8}^{*} = 1,e_{7,7}^{*} = 4,e_{7,8}^{*} = 7,e_{7,10}^{*} = 3, e_{8,9}^{*} = 2. \hfill \\ \end{gathered}$$

Therefore, from the definition of $NM$-Polynomial,

$$\begin{aligned} NM\left( {A_{4} ;x,y} \right) & = \mathop \sum \limits_{\delta \le i \le j \le \Delta } e_{i,j}^{*} x^{i} y^{j} \\ & = e_{2,4}^{*} x^{2} y^{4} + e_{2,5}^{*} x^{2} y^{5} + e_{3,5}^{*} x^{3} y^{5} + e_{3,6}^{*} x^{3} y^{6} + e_{3,7}^{*} x^{3} y^{7} \\ & \quad + e_{4,6}^{*} x^{4} y^{6} + e_{4,7}^{*} x^{4} y^{7} + e_{4,8}^{*} x^{4} y^{8} + e_{5,5}^{*} x^{5} y^{5} + e_{5,6}^{*} x^{5} y^{6} + e_{5,7}^{*} x^{5} y^{7} \\ & \quad + e_{5,8}^{*} x^{5} y^{8} + e_{5,9}^{*} x^{5} y^{9} + e_{6,6}^{*} x^{6} y^{6} + e_{6,7}^{*} x^{6} y^{7} + e_{6,8}^{*} x^{6} y^{8} + e_{7,7}^{*} x^{7} y^{7} \\ & \quad + e_{7,8}^{*} x^{7} y^{8} + e_{7,10}^{*} x^{7} y^{10} + e_{8,9}^{*} x^{8} y^{9} . \\ & = 9x^{2} y^{4} + x^{2} y^{5} + 6x^{3} y^{5} + 2x^{3} y^{6} + 2x^{3} y^{7} + 5x^{4} y^{6} \\ & \quad + 5x^{4} y^{7} + 3x^{4} y^{8} + x^{5} y^{5} + x^{5} y^{6} + x^{5} y^{7} + 3x^{5} y^{8} + x^{5} y^{9} + 24x^{6} y^{6} \\ & \quad + 14x^{6} y^{7} + x^{6} y^{8} + 4x^{7} y^{7} + 7x^{7} y^{8} + 3x^{7} y^{10} + 2x^{8} y^{9} . \\ \end{aligned}$$

Hence the result.

#### **Theorem 14**

* Let* $A_{4} \left( {V,E} \right)$ *be the isomorphic molecular graph of Analog* $A_{4}$, *then*.

1. $$M_{1} \left( {A_{4} } \right) = 492, NM_{1} \left( {A_{4} } \right) = {11}0{4}$$
2. $$M_{2} \left( {A_{4} } \right) = 631, NM_{2} \left( {A_{4} } \right) = {33}0{3}$$
3. $$mM_{2} \left( {A_{4} } \right) = 19.8055, NmM_{2} \left( {A_{4} } \right) = 3.8092$$
4. $${\text{ReZ}}G_{3} \left( {A_{4} } \right) = 3508,ND_{3} \left( {A_{4} } \right) = 42374$$
5. $$F\left( {A_{4} } \right) = 1384,NF\left( {A_{4} } \right) = {6948}$$
6. $$SDD\left( {A_{4} } \right) = 218.4166, ND_{5} \left( {A_{4} } \right) = {2}0{5}.{5975}$$
7. $$H\left( {A_{4} } \right) = 38.5857,NH\left( {A_{4} } \right) = {17}.{5818}$$
8. $$I\left( {A_{4} } \right) = 116.4476,NI\left( {A_{4} } \right) = {267}.{811}0$$

The computed indices values from above theorems are summarized in Tables 2 and 3.

**Table 2:** Antifungal drugs with topological indices values based on degree.

Drugs	$M_{1}$	$M_{2}$	$mM_{2}$	*ReZ* $G_{3}$	$F$	$SDD$	$H$	$I$
Posaconazole	422	630	30.0833	3984	1332	154.1665	24.9119	102.2047
Isavuconazole	304	489	5.4375	3314	1036	103.9166	15.0166	73.4166
Amphotericin B	462	597	17.2083	3328	1300	201.4166	30.6857	109.9142
Analog $A_{1}$	564	767	18.4305	4496	1654	233.3332	40.5190	134.8309
Analog $A_{2}$	536	708	18.4166	4018	1534	227.3333	40.0095	127.8738
Analog $A_{3}$	500	641	18.6527	3542	1394	218.7499	42.0523	119.0976
Analog $A_{4}$	492	631	19.8055	3508	1384	218.4166	38.5857	116.4476

**Table 3:** Antifungal drugs with topological indices values based on Neighbourhood degree-sum.

Drugs	$NM_{1}$	$NM_{2}$	$NmM_{2}$	$ND_{3}$	$NF$	$ND_{5}$	$NH$	$NI$
Posaconazole	993	3540	1.818	53,700	7425	150.505	10.723	241.978
Isavuconazole	706	2679	1.032	43,262	5652	101.005	6.5381	171.344
Amphotericin B	1036	3103	3.582	39,818	6518	193.077	16.450	251.269
Analog $A_{1}$	1284	4119	3.767	56,934	8602	223.841	18.338	313.053
Analog $A_{2}$	1198	3650	3.802	47,268	7618	216.701	18.219	291.757
Analog $A_{3}$	1124	3351	3.860	42,724	7022	208.939	17.862	272.734
Analog $A_{4}$	1104	3303	3.809	42,374	6948	205.597	17.581	267.811

## Results and discussion

### Applicability domain (AD) of QSPR/QSTR graph models

The AD of the QSPR/QSTR analysis for the drugs investigated in this research, including Posaconazole, Isavuconazole, Amphotericin B, and their analogs (A₁ to A₄), was evaluated based on structural and descriptor spaces. A descriptor-based approach was employed to establish the AD, focusing on the computed topological indices, including both degree-based and neighborhood degree-sum-based indices. These indices values, as presented in Tables 2 and 3, were determined to fall within the range of molecular descriptors, thereby ensuring the reliability and relevance of the model predictions within defined chemical and biological boundaries.

The following procedures were implemented for statistical validation to ensure the robustness of the AD.

#### Leverage analysis

The leverage value for each drug was computed to assess the influence of individual compounds on the regression models. A drug’s predictions were deemed reliable if its leverage value was below the critical threshold h* = 3(*p* + 1)/*n*, where *p* is the number of descriptors and *n* is the sample size. The leverage value ($h_{i}$) for each drug is calculated from the mathematical expression $h_{i} = X_{i}^{T} \left( {X^{T} X} \right)^{ - 1} X_{i}$ where, $X$ is the matrix of topological descriptors based on degree and neighborhood degree sum based indices (Tables 2 and 3), $X_{i}$ is the vector of indices for the *i*th drug, $X^{T}$ is the transpose of the matrix $X$ and $X_{i}^{T}$ is the transpose of the vector $X_{i}$. The leverage values of drugs are summarized in Table 4 which are computed through a python programming.

**Table 4:** Leverage values of antifungal drugs.

Drugs	Leverage value (h)
Posaconazole	0.3936
Isavuconazole	0.7508
Amphotericin B	1.8776
Analog $A_{1}$	0.5608
Analog $A_{2}$	− 0.7955
Analog $A_{3}$	0.3756
Analog $A_{4}$	0.9351

From Table 4, we observed that the computed leverage values ($h_{i}$) were below the critical threshold (h* = 7.2857). This means that no drugs were outlier in terms of leverage. This analysis ensures that the dataset is balanced for further modelling and statistical analysis.

The AD boundaries were defined by the combination of the descriptor ranges, leverage thresholds, and structural similarity measures. The dataset used for QSPR analysis to validate the properties consists of the drugs with well-characterized pharmacokinetic and toxicity properties, which ensures robust coverage of the relevant chemical space. The leverage analysis indicated that all drugs included in the research fall within acceptable boundaries, affirming the reliability of the predictions.

### Endpoint ranges and data pre-processing

In this section a detailed explanation that covers the endpoint range, pre-processing steps, criteria, normalization techniques, and handling of missing values in this article are provided. In Table 5, the ranges for ADMET and toxicity parameters based on the observed dataset are summarized.

**Table 5:** Endpoint ranges for ADMET and toxicity properties and their interpretations.

Properties	Range in dataset	Interpretation
WS	− 3.584 to − 2.658	Higher values (closer to 0) indicate better water solubility
Caco-2	− 1.236 to 1.145	Positive values denote high intestinal absorption; negative values indicate poor absorption
FU	0.148 to 0.666	Higher fraction unbound (FU) values suggest better bioavailability of the drug
BBB	− 2.872 to − 1.152	Higher (closer to 0) values suggest higher likelihood of crossing the blood–brain barrier
TC	− 1.701 to 0.29	Negative values indicate fewer toxic compounds; higher values correlate with greater toxicity risk
MTD	− 0.289 to 0.875	Maximum tolerated dose, where higher values indicate safer dosing ranges
CT	0.068–3.793	Cardiotoxicity scores, where higher values suggest a greater risk of adverse cardiovascular effects
LD_50_	0.762–3.107	A higher LD_50_ value indicates a lower acute toxicity, meaning the compound is safer at higher doses

The ADMET and toxicity properties of antifungal drugs reported in this article are considered based on their 80% availability. These ADMET properties are normalized using min–max scaling technique to ensure the consistent ranges. The ADMET properties reported are already pre-processed dataset, which are taken from the webserver pkCSM, (https://biosig.lab.uq.edu.au/pkcsm/prediction) and hence no interpolation is required for the other ADMET parameters. The drugs reported in this article for QSPR/QSTR analysis had complete ADMET profile after pre-processing. This ensures that the antifungal drugs reported in this article have consistent and reliable data for QSPR/QSTR analysis.

### QSPR and QSTR analysis of drugs through linear regression

**Fig. 10:**
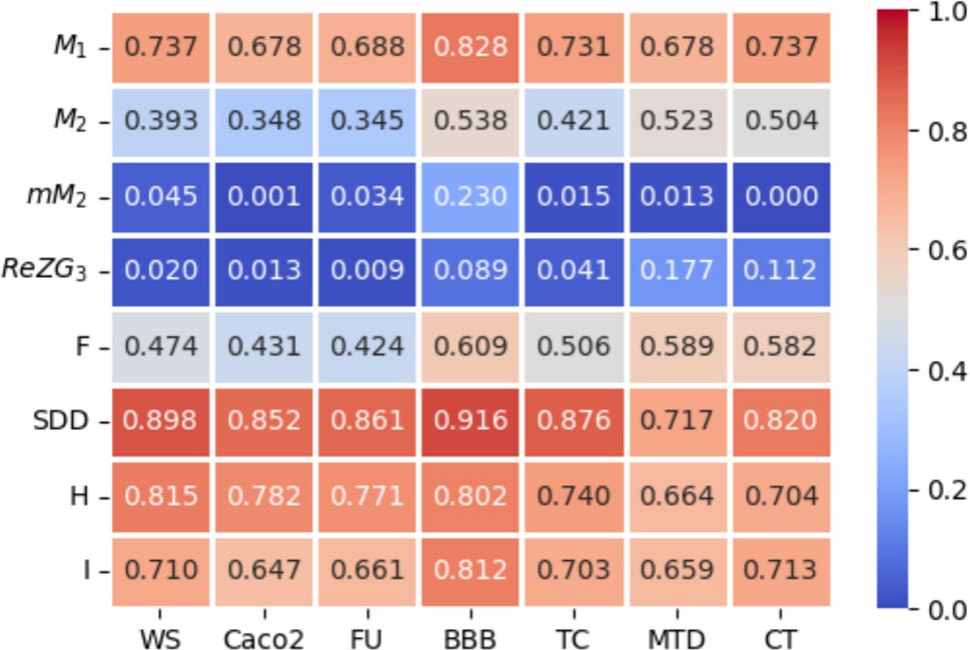
Heatmap for $R^{2}$ between degree-based indices and ADMET properties.

**Fig. 11:**
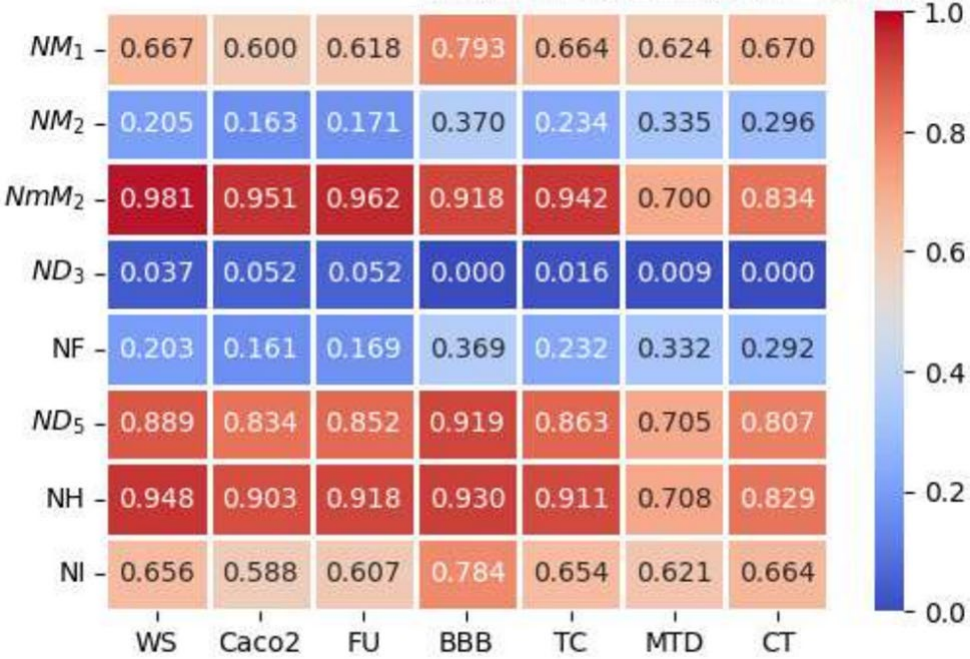
Heatmap for $R^{2}$ between neighborhood degree sum-based indices and ADMET properties.

The QSPR and QSTR analysis is considered between the computed topological indices and the ADMET and toxicity properties of antifungal drugs viz., Amphotericin B, Posaconazole, Isavuconazole, and analogs of Amphotericin B. The ADMET properties considered for QSPR analysis are namely Water solubility (WS), Caco2 permeability (Caco2), Fraction unbound (FU), BBB permeability (BBB), Total clearance (TC), Maximum tolerated dose (MTD) and Chronic toxicity (CT). The ADMET values of the abovementioned antifungal drugs are represented in Table 6, which are taken from the webserver pkCSM, (https://biosig.lab.uq.edu.au/pkcsm/prediction). In QSPR analysis, regression models play a crucial role, particularly when dealing with topological indices. They enable the assessment of the relationship between a chemical’s structure and its properties. The Statistical Package for the Social Sciences (SPSS) software was used to perform the regression analysis. A linear regression is considered for QSPR analysis to predict the ADMET properties, since this regression shows less standard error of estimation and high coefficient value when compared to quadratic and cubic regressions. The linear regression equation considered in the QSPR & QSTR analysis is

$$P = a + b\left( {TI} \right)$$

where $P$ represents the ADMET property as dependent variable, $a$ and $b$ are constant, and the $TI^{ }$ denotes the topological indices as independent variables in linear regression equation. The statistical parameters $R^{2}$ denote the coefficient of determination, $R^{ }$ is the correlation coefficient, $F$ denote the value of $F$-ration test and $SE$ is the standard error. For goodness of fit max ($R^{2}$), max ($R$), max ($F$), min ($SE$) values are considered for this model. The squared correlation coefficient value ($R^{2}$) determined between indices and the ADMET characteristics of antifungal drugs are depicted in Figs. 10 and 11. The best fitting and predictable linear regression equations having max ($R^{2}$) value are represented in Tables 7 and 8 and it is noted that the ADMET characteristics hold great significance since $R^{ } > 0.8$ and p-value is less than 0.05.

**Table 6:** Antifungal drugs and their ADMET values (WS, Caco-2, FU, BBB, TC, MTD, CT).

Drugs	WS	Caco2	FU	BBB	TC	MTD	CT
Posaconazole	− 3.368	1.145	0.251	− 2.122	0.071	0.875	0.068
Isavuconazole	− 3.584	1.072	0.148	− 1.152	0.29	0.627	0.364
Amphotericin B	− 2.695	− 1.035	0.661	− 2.872	− 1.624	0.264	2.819
Analog $A_{1}$	− 2.789	− 1.063	0.585	− 2.815	− 1.689	− 0.289	3.793
Analog $A_{2}$	− 2.737	− 1.08	0.588	− 2.726	− 1.449	− 0.098	3.758
Analog $A_{3}$	− 2.658	− 1.171	0.666	− 2.81	− 1.295	0.189	2.355
Analog $A_{4}$	− 2.746	− 1.236	0.61	− 2.795	− 1.701	0.017	3.282

**Table 7:** The statistical parameters for the highly correlated degree-based indices.

Linear regression equation	$R^{2}$	$R^{ }$	F	S. E	*P*-value
WS = − 4.38 + 0.0074 ($SDD$)	0.898	0.947	43.868	0.131	0.001
Caco2 = 3.61–0.02 ($SDD$)	0.852	0.923	28.712	0.459	0.003
FU = − 0.29 + 0.0041 ($SDD$)	0.861	0.928	30.886	0.086	0.003
BBB = 0.0066–0.0128 ($SDD$)	0.916	0.957	54.862	0.201	0.001
TC = 2.22–0.0169 ($SDD$)	0.876	0.936	35.362	0.331	0.002
MTD = 1.63–0.0072 ($SDD$)	0.717	0.847	12.667	0.238	0.016
CT = − 3.34 + 0.0294 ($SDD$)	0.820	0.906	22.839	0.717	0.005

**Table 8:** The statistical parameters for the highly correlated neighbourhood degree sum-based indices.

Linear regression equation	$R^{2}$	$R^{ }$	F	S. E	P-value
WS = − 3.92 + 0.3174 ($NmM_{2}$)	0.981	0.991	261.486	0.056	0.000
Caco2 = 2.33–0.9095 ($NmM_{2}$)	0.951	0.975	97.511	0.263	0.000
FU = − 0.05 + 0.1771 ($NmM_{2}$)	0.962	0.981	127.966	0.045	0.000
BBB = − 0.47–0.1326 ($NH$)	0.930	0.964	66.443	0.184	0.000
TC = 1.15–7147 ($NmM_{2}$)	0.942	0.970	80.499	0.228	0.000
MTD = 1.35–0.0742 ($NH$)	0.708	0.841	12.102	0.242	0.018
CT = − 1.39 + 1.2085 ($NmM_{2}$)	0.834	0.913	25.173	0.689	0.004

The best suited indices based on degree and neighbourhood degree sum for predicting the ADMET properties of antifungal drugs in the QSPR linear regression analysis are.

(i). The Symmetric Division degree index $\left( {SDD} \right)$ provides excellent correlation coefficient for Water solubility (WS), Caco2 permeability (Caco2), Fraction unbound (FU), BBB permeability (BBB), Total clearance (TC), Maximum tolerated dose (MTD) and Chronic toxicity (CT).
(ii). The Neighbourhood second modified Zagreb index ($$NmM_{2} )$$ is the best suitable index to predict Water solubility (WS), Caco2 permeability (Caco2), Fraction unbound (FU), Total clearance (TC), and Chronic toxicity (CT).
(iii). The Neighbourhood Harmonic index $\left( {NH } \right)$ is the best predictor index for BBB permeability (BBB) and Maximum tolerated dose (MTD).

**Fig. 12:**
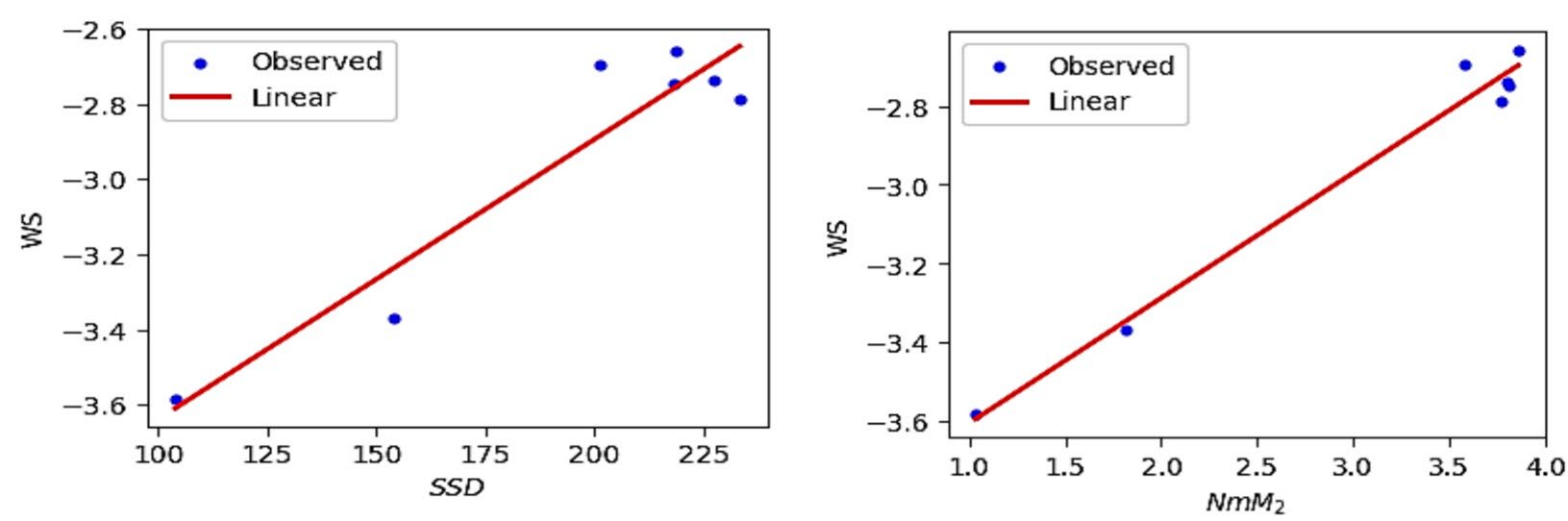
Linear plot of WS with $SDD$ index and $NmM_{2}$.

**Fig. 13:**
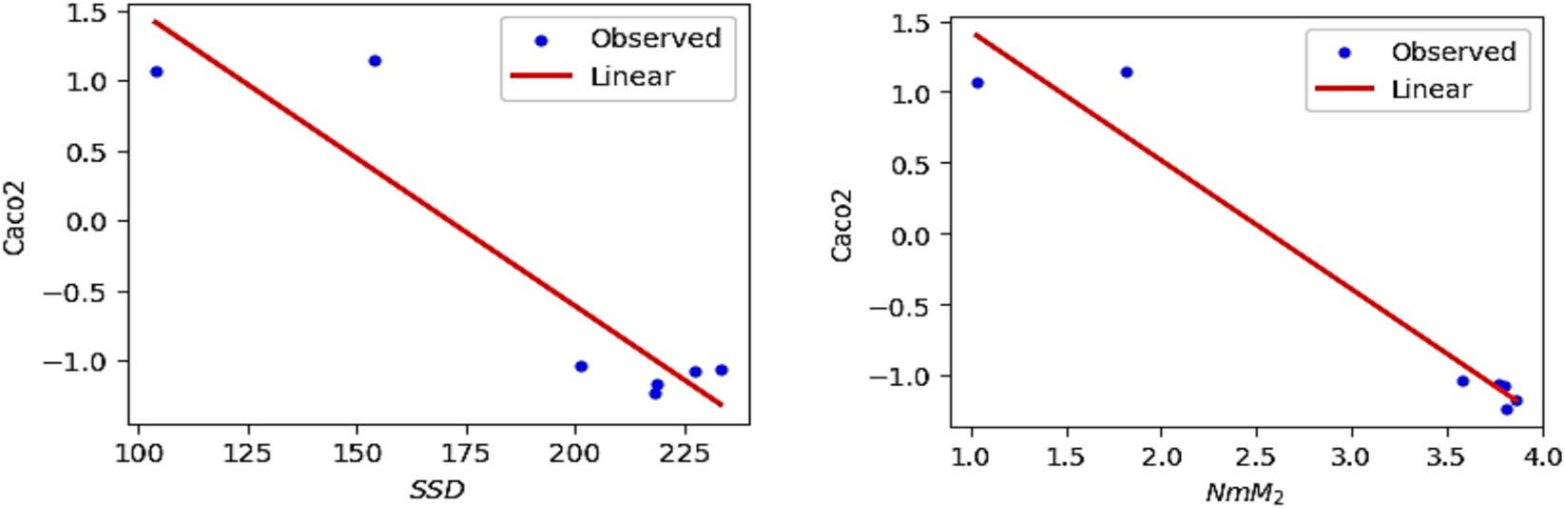
Linear plot of Caco2 with $SDD$ index and $NmM_{2}$.

**Fig. 14:**
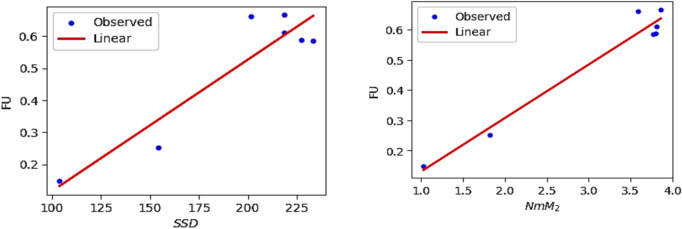
Linear plot of FU with $SDD$ index and $NmM_{2}$.

**Fig. 15:**
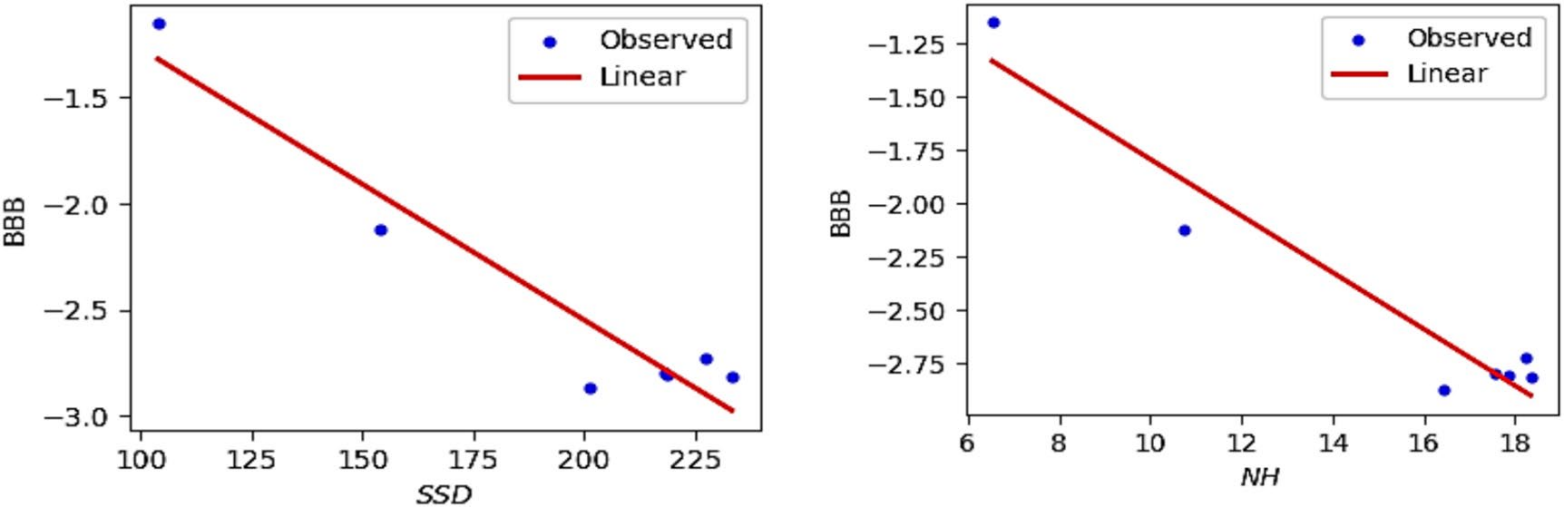
Linear plot of BBB with $SDD$ index and $NH$.

**Fig. 16:**
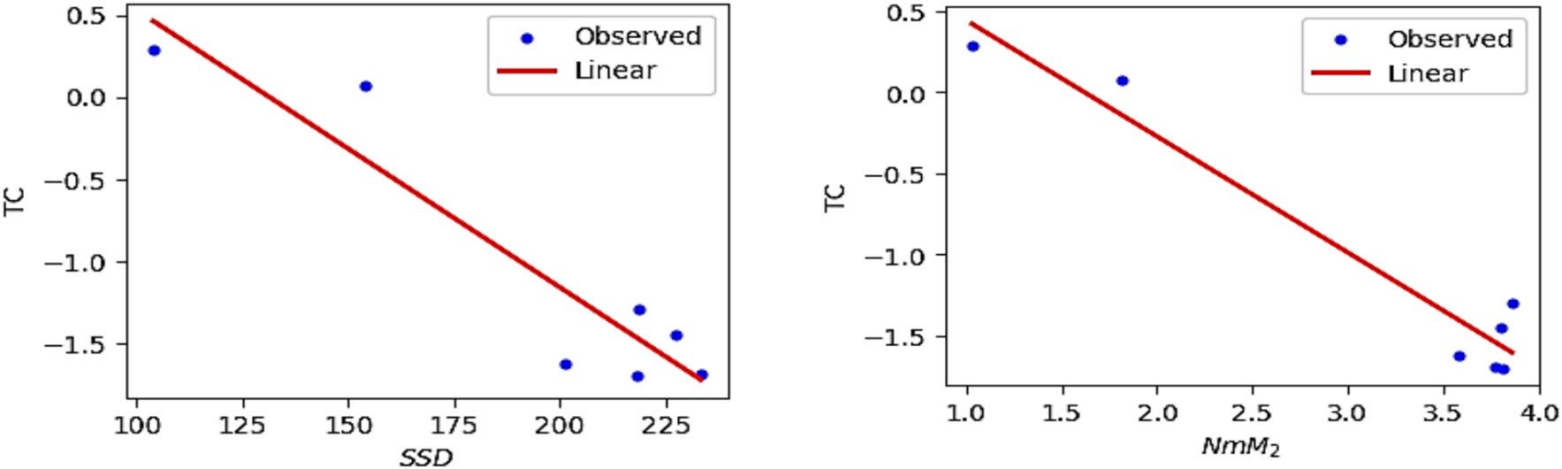
Linear plot of TC with $SDD$ index and $NmM_{2}$.

**Fig. 17:**
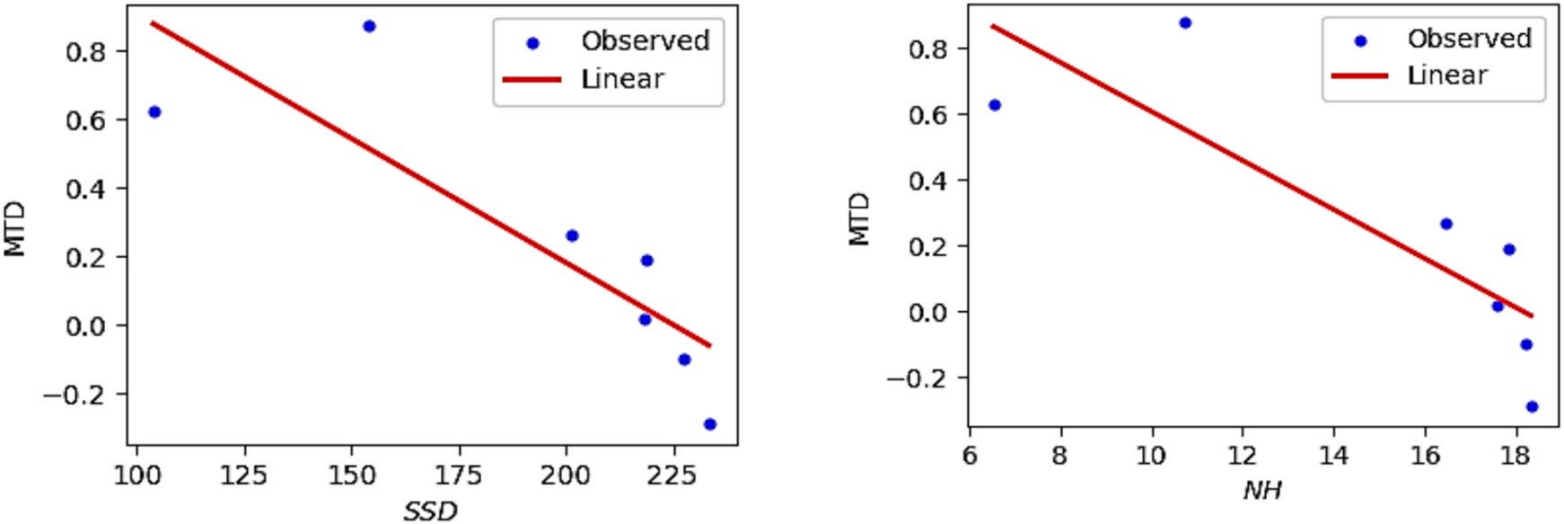
Linear plot of MTD with $SDD$ index and $NH$.

**Fig. 18:**
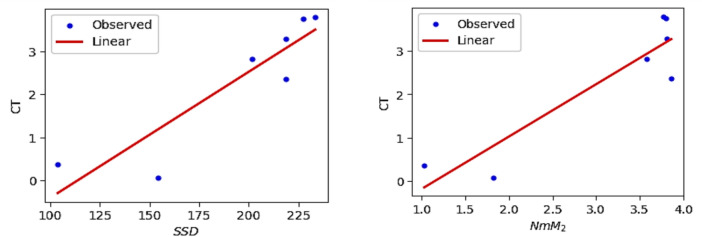
Linear plot of CT with $SDD$ index and $NmM_{2}$.

The Figs. 12, 13, 14, 15, 16, 17 and 18 from depict the linear regression plot for the best predictable indices for the ADMET characteristics of antifungal drugs.

**Fig. 19:**
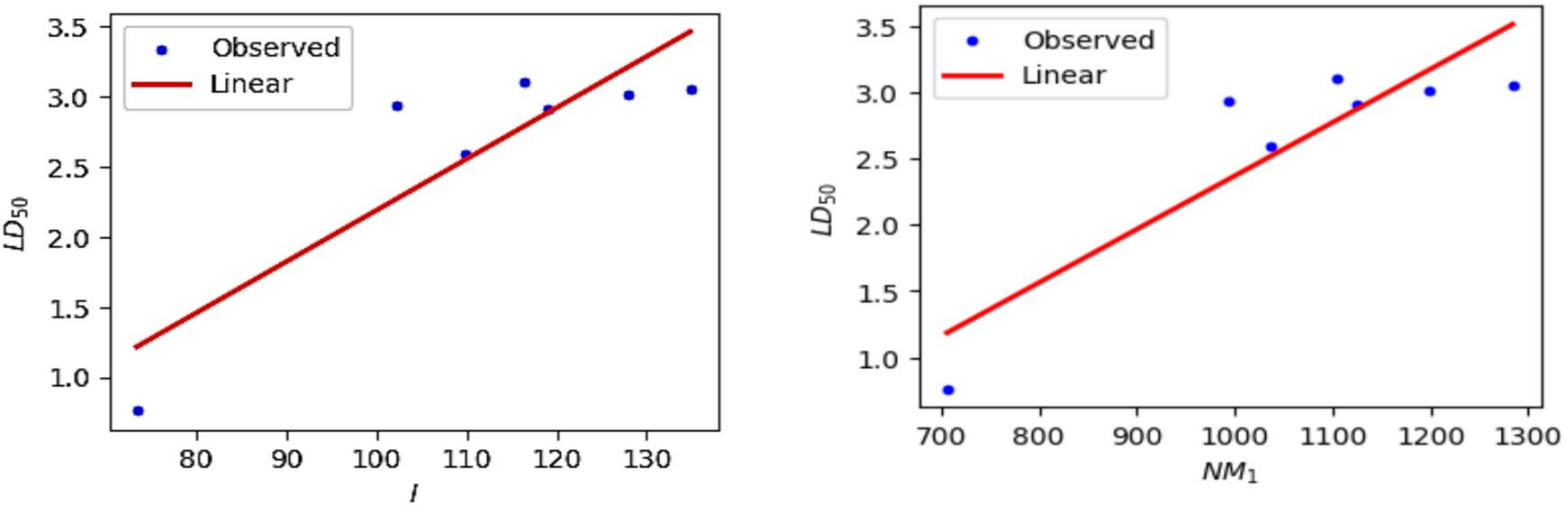
Linear regression curve for LD_50_ with $I$ and $NM_{1}$.

**Fig. 20:**
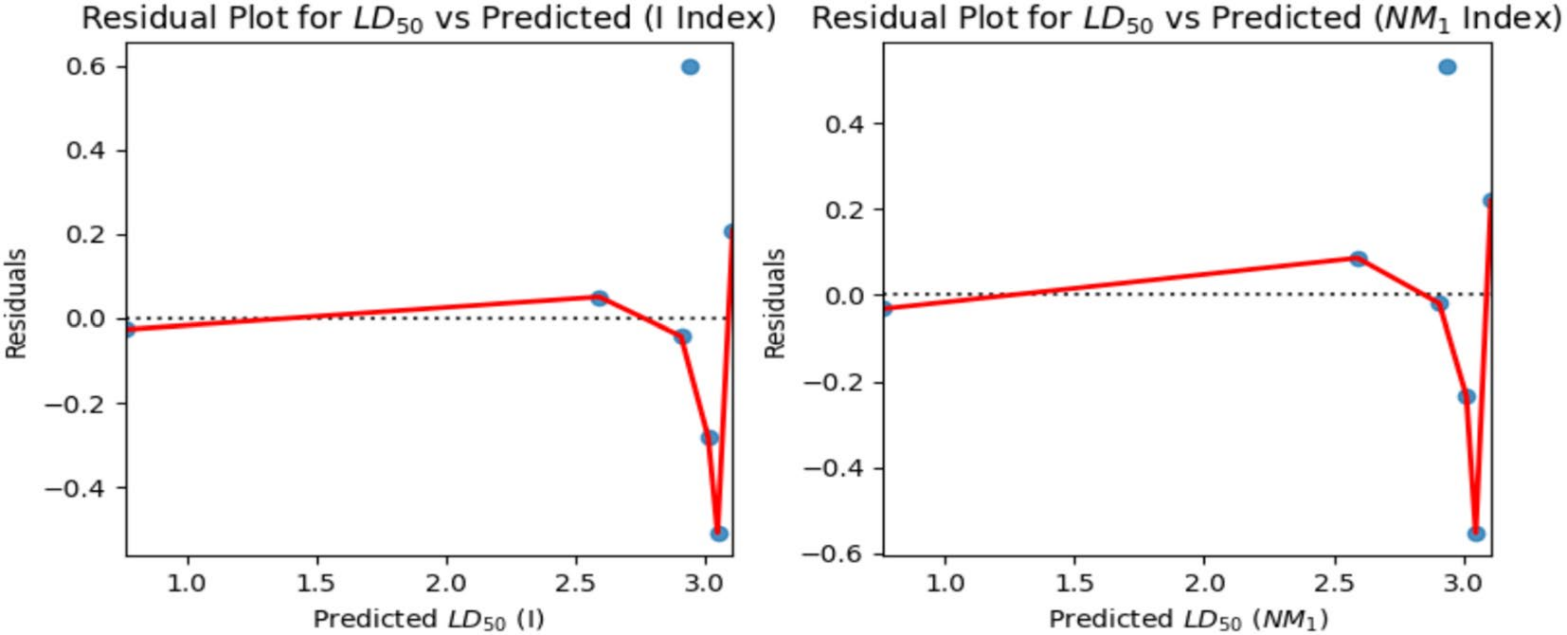
Residual plots for LD_50_ with $I$ and $NM_{1}$.

Similarly, the linear regression was applied to the 50% Lethal Dose value (LD_50_) and computed degree and neighbourhood degree sum-based indices. It was observed that the Inverse sum indeg index $$\left( I \right)$$ (R = 0.8778) and Third version Zagreb index ($$NM_{1} )$$ (R = 0.8887) exhibit a strong correlation with the computed LD_50_ values. The predicted LD_50_ values are obtained from the webserver pkCSM and the observed values derived using linear regression Eqs. (1) and (2) are presented in Tables 9 and 10. The Figs. 19 and 20 depict the linear regression plot and residual plot for LD_50_ with $$I$$ and $$NM_{1}$$ respectively.

1 $${\text{LD}}_{{{5}0}} = - {1}.{4654} + 0.0{365}(I)$$

2 $${\text{LD}}_{{{5}0}} = - {1}.{6543} + 0.00{4}0(NM_{1} )$$

**Table 9:** Predicted and observed LD_50_ activity value of Antifungal drugs based on Inverse sum indeg index $I\left( G \right)$.

Drugs	Predicted LD_50_	Observed LD_50_	Residuals
Posaconazole	2.938	2.26711	0.67089
Isavuconazole	0.762	1.21577	− 0.45377
Amphotericin B	2.592	2.54866	0.04334
Analog ${\varvec{A}}_{1}$	3.048	3.45862	− 0.41062
Analog ${\varvec{A}}_{2}$	3.012	3.20454	− 0.19254
Analog ${\varvec{A}}_{3}$	2.907	2.88404	0.02296
Analog ${\varvec{A}}_{4}$	3.107	2.78726	0.31974

**Table 10:** Predicted and observed LD_50_ activity value of Antifungal drugs based on Third version Zagreb index $NM_{1} \left( G \right)$.

Drugs	Predicted LD_50_	Observed LD_50_	Residuals
Posaconazole	2.938	2.33985	0.59815
Isavuconazole	0.762	1.18544	− 0.42344
Amphotericin B	2.592	2.51281	0.07919
Analog ${\varvec{A}}_{1}$	3.048	3.51036	− 0.46236
Analog ${\varvec{A}}_{2}$	3.012	3.16443	− 0.15243
Analog ${\varvec{A}}_{3}$	2.907	2.86678	0.04022
Analog ${\varvec{A}}_{4}$	3.107	2.78633	0.32067

### Cross validation of QSPR and QSTR linear regression analysis

The additional validations viz., Leave-one-out cross validation ($Q_{LOO}^{2}$), External validation ($Q_{EXT}^{2}$) and Five-fold cross-validation ($Q_{5 - Fold}^{2}$) are performed to claim the results obtained through QSPR/QSTR regression analysis are correct. These validations support the robustness and predictive power of the QSPR/QSTR linear regression analysis.

#### Leave-one-out cross validation ($Q_{LOO}^{2}$)

The $Q_{LOO}^{2}$ metric analyses the model’s performance by iteratively leaving out one data point at a time and evaluating how well the model predicts the left-out points. It is a reliable measure of model performance, particularly in small datasets, as it ensures that the analysis can generalize to unseen data. The calculated correlation coefficient ($Q_{LOO}^{2}$) and Root Mean Square Error of Cross Validation (RMSECV) for both degree-based and neighborhood degree sum-based indices across all properties through the mathematical expressions (3) and (4) are summarized in Table 11. The results in Table 11 show high $Q_{LOO}^{2}$ values and low RMSECV values indicating strong model fit and predictive power across most properties.

3 $$Q_{LOO}^{2} = 1 - \frac{{\mathop \sum \nolimits_{i = 1}^{n} \left( {y_{i}^{obs} - y_{i}^{{pred\left( {LOO} \right)}} } \right)^{2} }}{{\mathop \sum \nolimits_{i = 1}^{n} \left( {y_{i}^{obs} - \overline{y}_{ }^{obs} } \right)^{2} }}$$

4 $$RMSECV = \sqrt {\left( \frac{1}{n} \right)\mathop \sum \limits_{i = 1}^{n} \left( {y_{i}^{obs} - y_{i}^{{pred\left( {CV} \right)}} } \right)^{2} }$$

where $y_{i}^{obs} , y_{i}^{{pred\left( {LOO} \right)}} , \overline{y}_{ }^{obs} , y_{i}^{{pred\left( {CV} \right)}} ,$ and $n$ represent the observed (experimental) values, the predicted values obtained when that compound is excluded (using the model built on the remaining *n*-1 compounds), the mean of all observed values in the dataset, the predicted values obtained during the cross-validation process and the total number of compounds in the dataset respectively.

**Table 11:** $Q_{LOO}^{2}$ and $RMSECV$ value for both degree-based and neighborhood degree sum-based indices across all properties of antifungal drugs.

Properties	Degree based indices		Neighbourhood degree sum-based indices	
	$Q_{LOO}^{2}$	$RMSECV$	$Q_{LOO}^{2}$	$RMSECV$
WS	0.886442	0.110525	0.980806	0.047497
Caco2	0.825510	0.388223	0.948708	0.222429
FU	0.838068	0.072801	0.960930	0.037857
BBB	0.909003	0.170016	0.924880	0.155627
TC	0.858604	0.279999	0.937903	0.192356
MTD	0.603660	0.201129	0.584848	0.204627
CT	0.780947	0.605785	0.801437	0.581689
LD_50_	0.702499	0.371345	0.733785	0.355648

#### External validation ($Q_{EXT}^{2}$)

The external validation is a statistical metric used to evaluate the predictive power of a model on an independent external test set. This provides an estimate of the model’s performance on truly unseen data and further emphasizes its predictive capability. The correlation coefficient of external validation ($Q_{EXT}^{2}$) values computed using the mathematical expression (5) are reported in Table 12 for both sets of models (degree-based and neighborhood degree sum-based) were consistently high, reflecting good predictive accuracy and robustness, even on external test data.

5 $$Q_{EXT}^{2} = 1 - \frac{{\mathop \sum \nolimits_{i = 1}^{n} \left( {y_{i}^{obs} - y_{i}^{pred} } \right)^{2} }}{{\mathop \sum \nolimits_{i = 1}^{n} \left( {y_{i}^{obs} - \overline{y}_{ }^{obs} } \right)^{2} }}$$

where $y_{i}^{obs} , y_{i}^{pred} , \overline{y}_{ }^{obs} ,$ and $n$ represent the observed value for the *i*th sample in the test set, the predicted value for the *i*th sample from the model, the mean of the observed values in the training set and the number of samples in the external test set.

**Table 12:** $Q_{EXT}^{2}$ value for both degree-based and neighborhood degree sum-based indices across all properties of antifungal drugs.

Properties	Degree based indices	Neighbourhood degree sum-based indices
	$Q_{EXT}^{2}$	$Q_{EXT}^{2}$
WS	0.983777	0.997258
Caco2	0.975073	0.992673
FU	0.976867	0.994419
BBB	0.987000	0.989269
TC	0.979801	0.991129
MTD	0.943380	0.940693
CT	0.968707	0.971634
LD_50_	0.957500	0.961969

#### Five-fold cross-validation (${\varvec{Q}}_{{5 - {\varvec{Fold}}}}^{2}$)

The Five-fold cross-validation evaluates the stability of the model by splitting the dataset into 5 parts and performing multiple training and validation rounds. The $Q_{5 - Fold}^{2}$ values are computed using mathematical expression (6) and reported in Table 13 which support the model’s generalizability across multiple splits, showing consistent predictive performance. In particular, the $Q_{5 - Fold}^{2}$ values were consistently stable across all properties, reinforcing the robustness of the models.

6 $$Q_{5 - Fold}^{2} = 1 - \frac{{\mathop \sum \nolimits_{i = 1}^{n} \left( {y_{i}^{obs} - y_{i}^{pred} } \right)^{2} }}{{\mathop \sum \nolimits_{i = 1}^{n} \left( {y_{i}^{obs} - \overline{y}_{ }^{obs} } \right)^{2} }}$$

where $y_{i}^{obs} , y_{i}^{pred} , \overline{y}_{ }^{obs} ,$ and $n$ represent the observed (experimental) values, the predicted values for the *i*th sample from the model in its corresponding test fold, the mean of all observed values across the entire dataset, and the total number of compounds in the dataset respectively.

**Table 13:** $Q_{5 - Fold}^{2}$ value for both degree-based and neighborhood degree sum-based indices across all properties of antifungal drugs.

Properties	Degree based indices	Neighbourhood degree sum-based indices
	$Q_{5 - Fold}^{2}$	$Q_{5 - Fold}^{2}$
WS	0.752444	0.742117
Caco2	0.752513	0.742121
FU	0.752466	0.742093
BBB	0.752493	0.746917
TC	0.752484	0.742123
MTD	0.752443	0.746940
CT	0.752503	0.742126
LD_50_	0.772281	0.777716

#### Findings from cross-validation

(i). The $Q_{LOO}^{2}$ values for both degree based and neighborhood degree sum-based indices were all above 0.6, which supports the robustness and predictive power of our QSPR/QSTR graph models.
(ii). The $Q_{EXT}^{2}$ values of the both indices across all properties of antifungal drugs are above 0.9. This demonstrates excellent external validity.
(iii). $Q_{5 - Fold}^{2}$ values for these indices were also consistently high, averaging around 0.75–0.77 across various properties, indicating that the analysis generalize well even when tested on multiple folds of the data.

These metrics confirm that our QSPR/QSTR linear regression analysis exhibit strong generalizability and predictive performance across various indices and properties.

### Y-randomization test

The Y-randomization test is also conducted to confirm the robustness of our regression models for predicting ADMET properties. The purpose of this validation is to evaluate whether the observed relationship between the predicted and observed values is statistically significant or merely a result of chance. The Y-randomization test is performed using python programming and the output results are summarized in Tables 14 and 15 for both the indices.

**Table 14:** Comparison of original and scrambled R^2^ and MSE value of ADMET/ LD_50_ properties using degree-based indices through Y-randomization test.

Properties	Original R^2^	Original MSE	Mean Scrambled R^2^	Mean Scrambled MSE
WS	0.8864	0.0122	0.1585	0.0905
Caco2	0.8255	0.1507	0.1648	0.7214
FU	0.8381	0.0053	0.1793	0.0269
BBB	0.9090	0.0289	0.1726	0.2628
TC	0.8586	0.0784	0.1716	0.4593
MTD	0.6037	0.0405	0.1681	0.0849
CT	0.7809	0.3670	0.1591	1.4088
LD_50_	0.7021	0.1380	0.1792	0.3804

**Table 15:** Comparison of original and scrambled R^2^ and MSE value of ADMET/ LD_50_ properties using neighbourhood degree sum-based indices through Y-randomization test.

Properties	Original R^2^	Original MSE	Mean Scrambled R^2^	Mean Scrambled MSE
WS	0.9808	0.0023	0.1617	0.0985
Caco2	0.9487	0.0495	0.1627	0.8076
FU	0.9609	0.0014	0.1676	0.0305
BBB	0.9249	0.0242	0.1588	0.2712
TC	0.9379	0.0370	0.1757	0.4912
MTD	0.5848	0.0419	0.1689	0.0838
CT	0.8014	0.3384	0.1651	1.4227
LD_50_	0.7339	0.1264	0.1719	0.3934

#### Findings from Y-randomization test

(i). The original R^2^ values for all properties are high (close to or exceeding 0.8 for most properties), indicating that the analyses explain a large proportion of the variance in the observed data and the original MSE (Mean Standard Error) values are low, demonstrating high accuracy of the predictions.
(ii). The mean scrambled R^2^ values are consistently low (approximately 0.16–0.18 across all properties), reflecting weak or no relationship between predicted and scrambled observed values and the mean scrambled MSE values are significantly higher compared to the original MSE, indicating poor analysis when the observed values are randomized.
(iii). The significant gap between the original R^2^ and mean scrambled R^2^ and the corresponding MSE values confirms that the predictive performance of the analysis is not due to random chance.

These findings demonstrate that the analyses have captured meaningful relationships between the computed descriptors and ADMET properties. Thus, the Y-randomization test confirms that the considered QSPR/QSTR linear regression analyses are statistically significant and capture meaningful relationships between the molecular descriptors and the ADMET/ LD_50_ properties of antifungal drugs considered in this research.

## Conclusion

In this article, the isomorphic molecular graphs of antifungal drugs, including Amphotericin B, Posaconazole, Isavuconazole, and analogs of Amphotericin B were analyzed to explore their ADMET and toxicity properties. The $$M$$-polynomial and $$NM$$ -polynomial were computed for the isomorphic molecular graphs of these drugs. Subsequently, various degree-based and neighborhood degree sum indices were derived from these polynomials using the edge partition method. The QSPR and QSTR analyses were conducted to evaluate the relationships between the computed topological indices and the ADMET and toxicity properties of the antifungal drugs using linear regression. The computed topological indices exhibit a notably strong correlation coefficient with the majority of the properties. In particular, the Symmetric Division degree index $$\left( {SDD} \right)$$ and the Neighbourhood second modified Zagreb index ( $$NmM_{2} )$$ showed the highest significant positive correlation coefficients for most of the pharmacokinetic properties. Additionally, validation methods such as Leave-one-out cross validation ($$Q_{LOO}^{2}$$), External validation ($$Q_{EXT}^{2}$$) and Five-fold cross-validation ($$Q_{5 - Fold}^{2}$$) were performed to ensure the reliability of the results obtained through QSPR/QSTR regression analysis. These validations confirmed the robustness and predictive power of the QSPR/QSTR linear regression models. Furthermore, the Y-randomization test was conducted, verifying that the QSPR/QSTR linear regression analyses were statistically significant and captured meaningful relationships between the molecular descriptors and the ADMET/ LD_50_ properties of antifungal drugs considered in this research. The validation metrics confirm that the QSPR/QSTR linear regression analysis demonstrates strong generalizability and predictive performance across various indices and properties. Therefore, the work presented in this article could aid pharmaceutical industries in predicting the properties of antifungal drugs without the need for experimental testing.

## Data Availability

The datasets generated and/or analyzed during this study are available from publicly accessible repositories. The chemical data are accessible in the ChemSpider repository (https://www.chemspider.com/) and the pkCSM platform (https://biosig.lab.uq.edu.au/pkcsm/prediction). Chemical structures of the drugs were obtained from the National Centre for Biotechnology Information (NCBI) PubChem database (https://pubchem.ncbi.nlm.nih.gov/). The Statistical Package for the Social Sciences (SPSS) software (https://www.ibm.com/spss) was used for data analysis.
